# RegSNPs-intron: a computational framework for predicting pathogenic impact of intronic single nucleotide variants

**DOI:** 10.1186/s13059-019-1847-4

**Published:** 2019-11-28

**Authors:** Hai Lin, Katherine A. Hargreaves, Rudong Li, Jill L. Reiter, Yue Wang, Matthew Mort, David N. Cooper, Yaoqi Zhou, Chi Zhang, Michael T. Eadon, M. Eileen Dolan, Joseph Ipe, Todd C. Skaar, Yunlong Liu

**Affiliations:** 1 Center for Computational Biology and Bioinformatics, Indiana University School of Medicine, Indianapolis, IN 46202 USA; 2 Department of Medical & Molecular Genetics, Indiana University School of Medicine, 410 West 10th Street, Suite 5000, Indianapolis, IN 46202 USA; 3 Division of Clinical Pharmacology, Department of Medicine, Indiana University School of Medicine, 950 W Walnut St, Suite 419, Indianapolis, IN 46202 USA; 4 Institute of Medical Genetics, Cardiff University, Heath Park, Cardiff, CF14 4XN UK; 5 Institute for Glycomics and School of Informatics and Communication Technology, Griffith University, Parklands Dr., Southport, QLD 4215 Australia; 6 Division of Nephrology, Department of Medicine, Indiana University School of Medicine, Indianapolis, IN 46202 USA; 7 Section of Hematology/Oncology, Department of Medicine, University of Chicago, Chicago, IL 60637 USA

**Keywords:** Intron, Single nucleotide polymorphism, RNA splicing, Computational biology, Bioinformatics, Disease pathogenesis, Random forest, Prediction model, High-throughput screening assay

## Abstract

Single nucleotide variants (SNVs) in intronic regions have yet to be systematically investigated for their disease-causing potential. Using known pathogenic and neutral intronic SNVs (iSNVs) as training data, we develop the RegSNPs-intron algorithm based on a random forest classifier that integrates RNA splicing, protein structure, and evolutionary conservation features. RegSNPs-intron showed excellent performance in evaluating the pathogenic impacts of iSNVs. Using a high-throughput functional reporter assay called ASSET-seq (ASsay for Splicing using ExonTrap and sequencing), we evaluate the impact of RegSNPs-intron predictions on splicing outcome. Together, RegSNPs-intron and ASSET-seq enable effective prioritization of iSNVs for disease pathogenesis.

## Background

Prior to the advent of genome-wide association studies (GWAS), research on the relationship between genetic variants and human disease had largely focused on non-synonymous single nucleotide variants (SNVs) located in protein-coding regions. However, next-generation sequencing has enabled discovery of non-coding variants in the human genome [1]. In fact, the vast majority (88%) of trait or disease-associated variant loci identified in GWAS are in non-coding regions (45% intronic and 43% intergenic) [2]. Compared with intergenic SNVs that can modulate gene expression by altering chromatin states and promoter or enhancer-associated activity [3, 4], intronic SNVs (iSNVs) mainly regulate biological activities by dysregulating mRNA splicing [5–8]. For instance, over 20,000 disease-causing iSNVs in the Human Gene Mutation Database (HGMD) have been documented to impact splicing, and most of these pathogenic variants are located close to splice-junction boundaries [9].

To date, many algorithms and computational models have been developed to annotate non-coding regulatory variants, including RegulomeDB, HeploReg, and others [10–12]. These tools annotate regulatory non-coding SNVs that are trait-associated or predict their impact on biological function by determining whether the variants overlap with known regulatory elements, such as transcription factor (TF) binding sites, enhancers or promoters, methylation sites, and other features. Annotations for these regulatory elements are sourced from databases including TRANSFAC and JASPAR [12–14], or with data provided by consortia like ENCODE [15, 16]. In addition to simply overlapping the variants with known functional annotation, many tools have been developed to decipher the characteristics of functional or neutral non-coding variants from large amounts of training data using various machine learning-based classification methods that integrate genomic or other annotations as features. These learned characteristics are then used to classify new functional variants from neutral ones. One widely used machine learning tool, CADD (Combined Annotation Dependent Depletion), predicts pathogenic variants via support vector machine (SVM) by combining annotations from multiple sources including sequence conservation, such as PhyloP; regulatory elements, such as transcription factor binding; and protein-level predictions, such as SIFT and PolyPhen [17]. Similar tools have achieved comparable function and performance by using different training datasets or machine learning algorithms. Such tools include DANN (a deep neural network algorithm) [18], GWAVA (a random forest classifier) [19], FATHMM-MKL (a multi-kernel learning approach) [20], and LINSIGHT (hybrid of a linear and probabilistic models) [21]. Importantly, the focus of these tools has been primarily on identifying the potential pathological impact of coding variants or non-coding variants in promoter and enhancer regions that regulate transcription. In fact, very few algorithms target on intronic variants and their impact on splicing regulation.

Intronic variants can impact alternative splicing by interfering with splice site recognition. For example, an intronic mutation near the 5′-splice site of exon 20 in the *IKBKAP* gene causes skipping of exon 20, resulting in malfunction of IKBKAP in 99.5% of familial dysautonomia (FD) cases [8, 22, 23]. Likewise, the intron 4 splice-donor site variant in the *adenomatous polyposis coli* (*APC*) gene causes skipping of exon 4, which can lead to colon cancer [24, 25]. In addition to iSNVs located in splice sites, a number of deep intronic mutations also contribute to disease, such as those recently described in the *GALNS* gene that cause the lysosomal disorder called Morquio A disease [26]. Additionally, iSNVs may alter the binding affinities of RNA-binding proteins (RBP) to cis-regulatory elements [5, 27, 28]. For example, a G to A substitution within an intronic-splicing enhancer downstream of exon 3 in the *growth hormone* (*GH1*) gene can cause familial isolated GH deficiency type II (IGHD II) by suppressing the binding of splicing factors [29–31]. While iSNVs are densely distributed in the genome, only a limited proportion have been investigated for associations with altered biological functions [5].

Owing to the large number of intronic variants detected in next-generation sequencing and their complex RNA splicing regulatory mechanisms, efficient bioinformatics algorithms are required to predict the potential impact of iSNVs and to prioritize them for functional studies. One algorithm, SPANR (Splicing-based Analysis of Variants) [32], was designed to evaluate how individual SNVs impacted splicing regulation by predicting the maximum change in the percentage of inclusion (dPSI) of nearby exons induced by the SNVs. It extracts 1393 genomic features around the SNVs and predicts potential splicing outcomes by training a neural network with RNA-seq data from 16 human tissues. However, SPANR was not designed to assess whether iSNVs resulted in deleterious phenotypes since it does not evaluate the impact of the resultant splicing change on protein function. Other ensemble-based tools reviewed in detail [12], including CADD [17], use machine learning approaches to predict pathogenic variants by combining annotations from multiple sources. However, these annotations do not include RNA splicing data and provide limited information on how splicing variants influence protein function. To this end, our earlier studies on small insertions/deletions (INDELs) [33, 34], alternatively spliced exons [35], and synonymous SNVs [36, 37] have indicated that simply substituting a stretch of amino acid residues does not necessarily imply altered protein function. Others have also reported that nucleotide substitutions can be bystander events, i.e., non-consequential to the phenotype [38]. Therefore, in order to consider the molecular implications of functional iSNVs, it is critical to integrate features that more accurately predict the effects of alternative-splicing events on protein structure and function.

In this study, we considered the impact of iSNVs on splicing regulation together with the variant-induced impact of alternatively spliced exons on protein-structure features. We extracted pathogenic iSNVs from the HGMD and randomly selected neutral iSNVs from the 1000 Genomes Project [9, 39]. Using these data, we developed an algorithm based on the random forest method to compute the disease-causing probabilities of iSNVs [40], which we have termed RegSNPs-intron. This algorithm was also tested on an independent dataset selected from the ClinVar database [41]. In addition, we designed a high-throughput functional reporter assay, ASSET-seq (ASsay for Splicing using ExonTrap and sequencing), to experimentally validate the effects of the predicted iSNVs on splicing regulation. As we demonstrate here, RegSNPs-intron and ASSET-seq can be used in tandem to prioritize and screen potential pathogenic iSNVs to gain better understanding of their roles in complex disease.

## Results

### Datasets

**Fig. 1:**
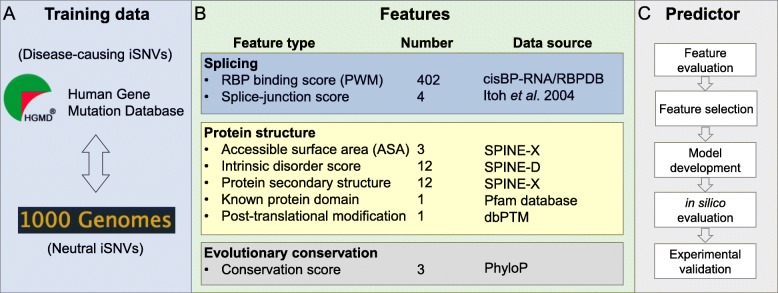
Workflow. **a** Data sources for model building. Pathogenic and neutral iSNVs were collected from HGMD and 1000 Genomes Project, respectively. **b** Three categories of features, namely RNA splicing, protein structure, and evolutionary conservation, were considered for the iSNVs. Data sources and the numbers of these features are listed on the side. For additional details for the data sources, refer to the “Methods” section. **c** Logic flow for prediction model development, as well as model evaluation and result validation

To compute the pathogenic probabilities of iSNVs, we constructed a training set by combining the manually curated pathogenic iSNVs in the HGMD and the putatively neutral iSNVs from the 1000 Genomes Project phase 3 (Fig. 1 and Additional file 1: Figure S1). The neutral iSNVs documented in the 1000 Genomes dataset were derived from genome sequencing data from 2500 individuals lacking obvious clinical phenotypes [1]. In order to minimize the false negatives in our training set, we only included iSNVs with minor allele frequency (MAF) greater than 10%. This selection resulted in 2438 pathogenic and 2,104,613 neutral iSNVs.

Since the proximity of an iSNV to the splice-junction site can impact the splicing outcome via different molecular mechanisms, we further divided the selected iSNVs into those proximal and distal to the splice-junction sites. For example, variants proximal to the splice-junction sites (on-ss) may directly interfere with spliceosome formation, while iSNVs distal from the junction sites (off-ss) may affect the binding of regulatory RNA-binding proteins (RBPs). Splice-junction sites were defined as the upstream 13-bp for 3′-acceptor sites and the downstream 7-bp for 5′-donor sites [42]. In total, there were 1865 on-ss and 573 off-ss pathogenic iSNVs and 3386 on-ss and 2,104,613 off-ss neutral iSNVs. These data revealed that pathogenic variants were found more frequently in regions proximal to splice-junction sites compared to neutral variants (Additional file 1: Figure S2). To avoid potential bias introduced by the varying distances of iSNVs from junction sites, we randomly selected 852 off-ss neutral iSNVs from the 1000 Genomes dataset by matching the distance distribution of the pathogenic HGMD variants. This ensured more balanced datasets with similar distance distributions between the pathogenic and neutral variants.

For each of the pathogenic and neutral on-ss and off-ss datasets, we randomly selected two thirds of the data for use as the training set to build a random forest classifier (Additional file 1: Figure S1). The remaining one third of the data were used as a test set. To further test the model performance, we also extracted pathogenic and neutral iSNVs from the ClinVar database as an independent test set [41], which included 121 on-ss and 51 off-ss pathogenic iSNVs and 167 on-ss and 883 off-ss neutral iSNVs (see the “Methods” section for details).

**Fig. 2:**
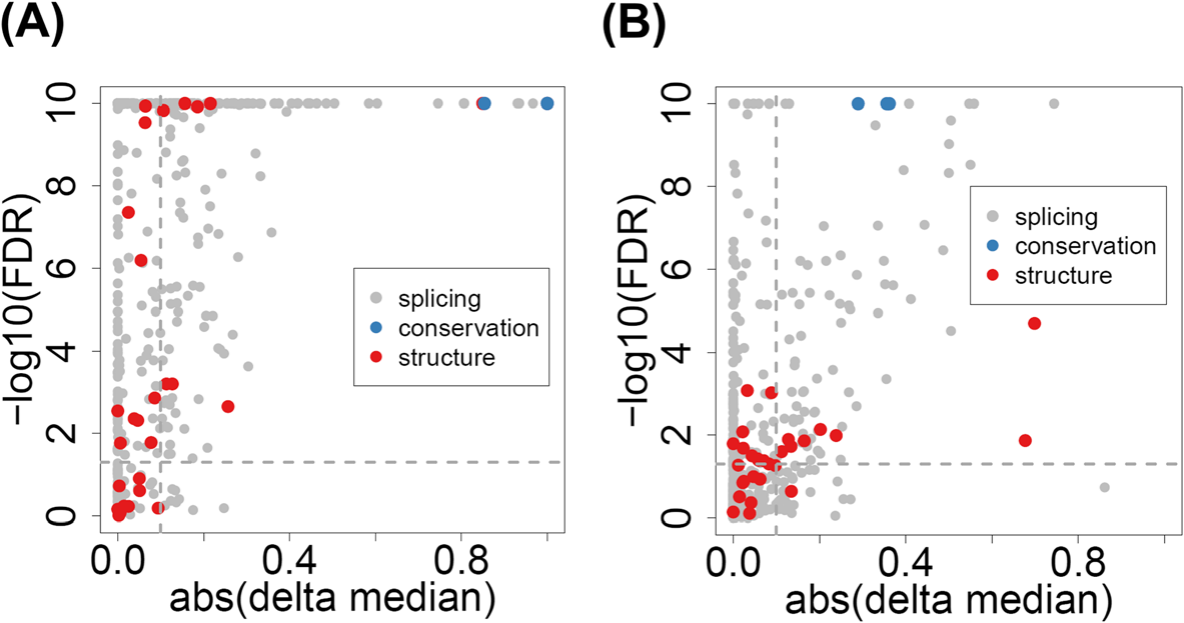
Feature evaluation. Significance of difference in feature scores between pathogenic and neutral iSNVs. **a** on-ss iSNVs. **b** off-ss iSNVs. Features were from three categories—splicing (gray), protein structure (red), and evolutionary conservation (blue). See also Additional file 2: Table S1 for more details. In the plot, every dot represents a feature. The *x*-axis is (the absolute value of) the feature’s delta median, calculated as the “median score of pathogenic iSNVs” minus the “median score of neutral iSNVs” of the particular feature. Calculations of feature scores were described in the “Methods” section. The *y*-axis is the significance value of the score difference based on Wilcoxon’s rank-sum test (*p* values adjusted by FDR and transformed by − log10, capped at 10^−10^ for display). Due to the large dynamic ranges of the feature values, the delta medians were rescaled to range [− 1, 1]: *x*’ = 2 × (*x* − min(*x*))/(max(*x*) − min(*x*)) − 1

In order to select features that optimally discriminate pathogenic and neutral iSNVs, we classified all features into three categories: (i) splicing features, characterizing how individual iSNVs affect splicing regulation; (ii) structural features, evaluating how the iSNV-induced inclusion/exclusion of alternatively spliced exons affects protein functions; and (iii) evolutionary conservation features, measuring the nucleotide base-wise conservation scores of 99 vertebrate genomes (Fig. 1). In these three categories, 360 of the 438 features showed significant power in separating the pathogenic and neutral on-ss iSNVs, whereas 194 of the 436 features separated the off-ss iSNVs based on the Wilcoxon rank-sum test with an adjusted *p* value < 0.05 (Fig. 2 and Additional file 2: Table S1).

### Disease-causing iSNVs affect alternative splicing

To evaluate the impact of an iSNV on splicing regulation, we considered two measures: (i) the splice-junction scores associated with proximal exons in both 5′ and 3′ directions, as well as the deviation of the junction score of the variant from the reference allele, and (ii) the iSNV-induced difference in RBP binding affinity. The splice-junction score was computed by position weighted matrices (PWMs) measuring sequence features around the canonical junction sites (see the “Methods” section for details) [42]. Higher scores are more likely to include the corresponding exons in the resulting mRNA. Our results showed that pathogenic iSNVs were more frequently associated with lower splice-junction scores. The median junction score of pathogenic on-ss iSNVs was 7.37 and 7.11 for donor and acceptor sites, respectively, compared to 7.95 and 7.84 for neutral iSNVs (adjusted *p* value 0.017 and 1.35 × 10^−7^, respectively) (Additional file 2: Table S1). For pathogenic off-ss iSNVs, the median junction scores were 7.63 and 7.95 for donor and acceptor sites, compared to 8.13 and 8.54 for neutral off-ss iSNVs (adjusted *p* value 0.018 and 9.0 × 10^−4^, respectively) (Additional file 2: Table S1). In addition to the junction scores, the deviation of the junction score resulting from each on-ss iSNV was calculated. The median deviation values for pathogenic iSNVs were − 2.96 and − 2.23 for donor and acceptor sites, respectively. The magnitude of these deviations was significantly larger than those of neutral iSNVs, which were 0.034 and − 0.059 for donor and acceptor sites, respectively (Additional file 1: Figure S3). The adjusted Wilcoxon rank-sum test *p* values for donor and acceptor sites were 1.63 × 10^−211^ and 1.28 × 10^−143^, respectively (Additional file 2: Table S1 and Additional file 1: Figure S3). These results indicate that pathogenic iSNVs are strongly associated with exon skipping.

To evaluate the impact of an iSNV on RBP binding, we computed both the magnitude and probability of binding score changes caused by the iSNVs for 201 RBPs with known PWMs. We found that pathogenic and neutral iSNVs showed significant differences in the binding of many RBPs. Generally, the pathogenic on-ss and off-ss iSNVs induced large binding score changes in 191 and 176 RBPs, respectively, compared to the respective neutral iSNVs. Specifically, 137 RBPs showed significant differences in iSNV-induced binding scores between on-ss pathogenic and neutral variants, whereas 43 RBPs showed significant differences between off-ss pathogenic and neutral variants (adjusted Wilcoxon rank-sum test *p* value < 0.05; Additional file 2: Table S1 and Additional file 1: Figure S4). Taken together, these findings provide strong evidence that pathogenic iSNVs affect splicing regulation and result in altered mRNA structures.

### Disease-causing iSNVs are associated with exons encoding functionally important protein domains

To evaluate the impact of iSNVs on protein function, we examined the structural features corresponding to potential alternatively spliced exons. We hypothesized that pathogenic iSNVs disrupt the splicing of exons that encode key protein structural domains. We captured the protein structural features for the closest neighboring exons of the iSNVs including intrinsic disorder score, secondary structure (e.g., alpha helix, beta sheet, or random coil), and solvent accessible surface areas (ASA) (Additional file 2: Table S1) [43, 44]. We also calculated the overlap percentage of the target exon with known protein domains, as well as the number of known post-translational modification sites within the exon-encoded protein domain (Additional file 2: Table S1) [45, 46].

We found that exons in proximity to pathogenic iSNVs were more likely to encode protein domains that had lower average disorder scores and contained longer structured regions, compared to exons proximal to neutral iSNVs (adjusted Wilcoxon rank-sum test *p* value 2.03 × 10^−11^ and 0.0497 for on-ss and off-ss iSNVs, respectively) (Additional file 2: Table S1 and Additional file 1: Figure S5). This result indicates that pathogenic iSNVs have a higher probability of being proximal to exons encoding structured regions. In addition, proximal exons to pathogenic iSNVs had significantly smaller average ASA scores (adjusted *p* value 2.90 × 10^−13^ and 0.0103 for on-ss and off-ss iSNVs, respectively), which indicates that they are more likely to encode regions in the protein core as opposed to regions on the protein surface. Moreover, the closest exons to pathogenic iSNVs encoded a significantly higher percentage of residues that overlapped with known protein domains (adjusted *p* value 1.91 × 10^−16^ and 1.98 × 10^−5^ for on-ss and off-ss iSNVs, respectively). Taken together, these results show that exons proximal to pathogenic iSNVs are more likely to encode functionally important protein regions. On the other hand, our analysis also suggests that protein structural features provide valuable information for prioritizing disease-causing iSNVs.

### Disease-causing iSNVs localize to conserved regions

Previous studies showed that evolutionary conservation is an important feature in assessing the disease-causing potential of SNVs [47, 48]. To determine whether an iSNV was evolutionarily conserved, we calculated the PhyloP 100-way conservation score of the iSNV locus, and the mean conservation score of a region flanking either side of the candidate iSNV by a length of 3 bp as well as 7 bp. Our results showed that pathogenic iSNVs had significantly higher PhyloP conservation scores than neutral iSNVs (Additional file 1: Figure S6). The median conservation score for on-ss pathogenic iSNV loci was 3.08, which was significantly higher than the score of − 0.03 for neutral on-ss iSNVs (adjusted Wilcoxon rank-sum test *p* value < 1 × 10^−300^). Likewise, the median score for off-ss pathogenic iSNV loci was 0.31, compared to − 0.28 for neutral off-ss iSNVs (adjusted *p* value 3.21 × 10^−38^). The large positive PhyloP scores for the pathogenic iSNV loci suggested that they evolved much more slowly than the neutral loci. Conservation scores for the 3-bp and 7-bp flanking regions were consistent with the iSNV loci scores. For the 3-bp flanking regions around the on-ss and off-ss pathogenic iSNVs, the respective medians of their mean conservation scores were 2.86 and 0.23, compared to 0.58 and − 0.07 for the neutral iSNVs (adjusted *p* values 1 × 10^−300^ and 5.47 × 10^−23^, respectively). Similar results were also found for the 7-bp flanking regions. These findings indicate that iSNVs at more conserved loci are more likely to be pathogenic.

### RegSNPs-intron model building, performance, and evaluation

Based on the splicing, protein structure, and evolutionary conservation features described above (Additional file 2: Table S1), random forest classifiers were built for on-ss and off-ss iSNVs, respectively. The models were built on the training set (two thirds of the original dataset), and their predictive powers were evaluated on the validation set (one third of the original dataset). Hyperparameters, such as number of trees and maximum depth, were optimized via the grid search with threefold cross-validation on the training set. For on-ss iSNVs, the random forest model contained 52 trees with the maximum depth of 13. For off-ss iSNVs, 59 trees with the maximum depth of 20 were built. The resulting on-ss and off-ss models constitute RegSNPs-intron.

**Fig. 3:**
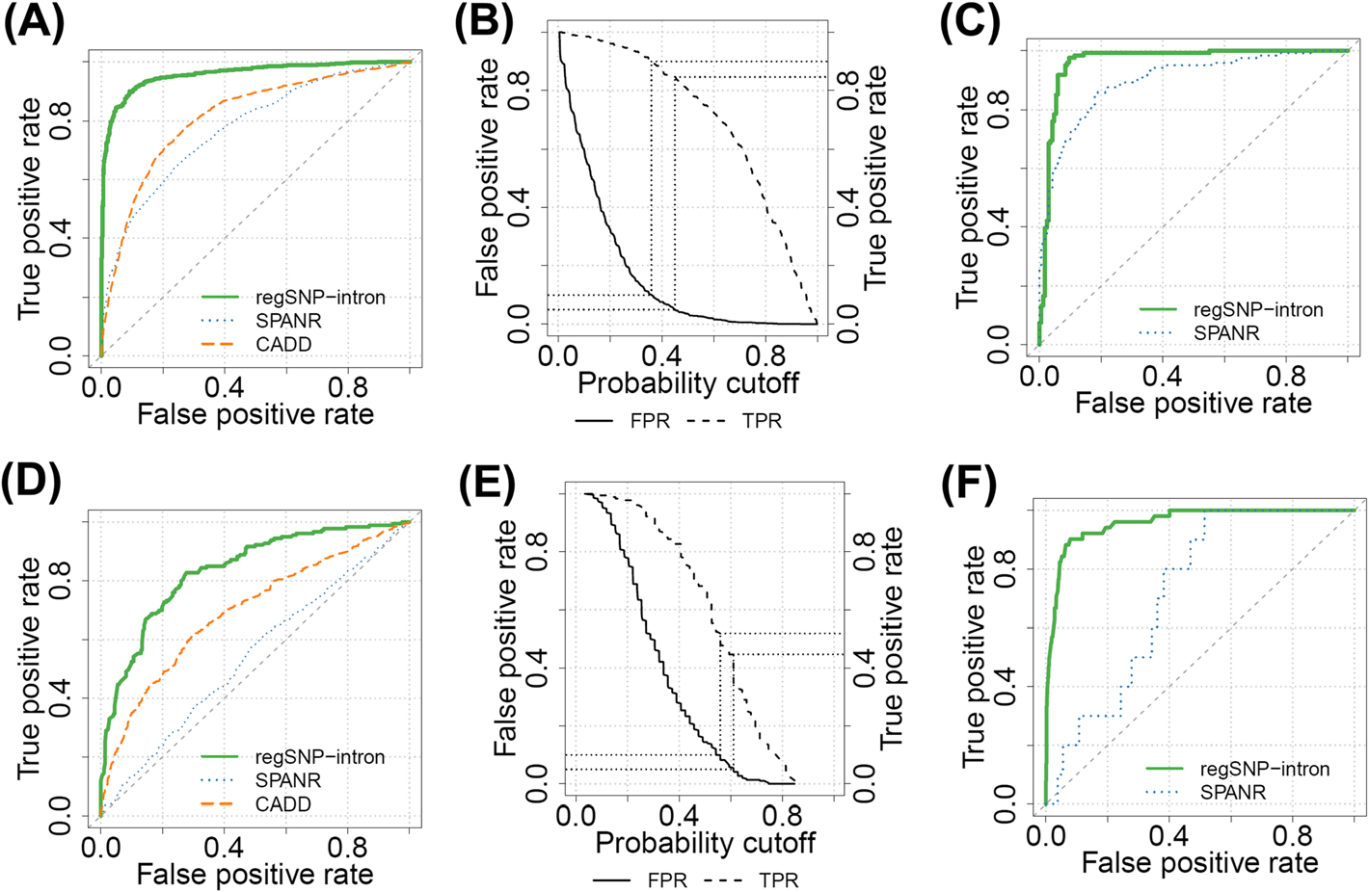
Model performance, evaluation, and analysis. **a**–**c** On-ss iSNVs. **a** Receiver operating characteristic curves (ROC) of RegSNPs-intron (solid green), SPANR (dotted blue), and CADD (dashed orange) on the validation set. **b** Selected probability cutoffs based on the false-positive rate (solid) and true-positive rate (dashed). The two dotted lines indicate FPR = 0.05 and 0.1, respectively. iSNVs with FPR < 0.05 are *Damaging*, those with 0.05 ≤ FPR < 0.1 are *Possibly Damaging*, and iSNVs with FPR ≥ 0.1 are *Benign*. **c** ROC of RegSNPs-intron (solid green) and SPANR (dotted blue) on the independent ClinVar test set. CADD was excluded since ClinVar was used in its model training. **d**–**f** Off-ss iSNVs, same as **a**–**c**

Based on the validation set (one third of the original dataset, not used in model training), RegSNPs-intron reached an AUROC (area under the receiver operating characteristic curve) of 0.96 and a Matthews correlation coefficient (MCC) of 0.79 for on-ss iSNVs and outperformed both SPANR and CADD (AUROCs 0.77 and 0.81, respectively, Fig. 3a). For off-ss iSNVs, RegSNPs-intron AUROC was 0.84 (MCC 0.52), compared to SPANR and CADD AUROCs of 0.54 and 0.69, respectively (Fig. 3d). Here, we also calculated the AUPR (area under the precision recall curve) as the numbers of pathogenic and neutral iSNVs might be imbalanced. For on-ss iSNVs, RegSNPs-intron had an AUPR of 0.94, compared to 0.70 and 0.71 for SPANR and CADD, respectively (Additional file 1: Figure S7A). The AUPR given by RegSNPs-intron for off-ss iSNVs was 0.77, compared to SPANR and CADD AUPRs of 0.47 and 0.62, respectively (Additional file 1: Figure S7C). Our results suggest that inclusion of protein structural features encoded by the proximal exons, which are not used by either SPANR or CADD, significantly increases the performance of RegSNPs-intron in predicting variant pathogenicity.

To further evaluate the predictive powers of the features related to splicing, structure, and conservation, we built separate models based on features from each of these three categories. For on-ss iSNVs, the AUROCs for splicing, structure, and conservation features were 0.92, 0.72, and 0.92, respectively; for off-ss iSNVs, the AUROCs were 0.75, 0.63, and 0.68, respectively (Additional file 1: Figure S8). These results demonstrate that each category of features provides important information in model prediction, and thus, the combination of all three categories yields the highest performance.

To control the false-positive rate (FPR) of the prediction results, we reported the iSNVs with FPR < 0.05 as *Damaging*, iSNVs with 0.05 ≤ FPR < 0.1 as *Possibly Damaging*, and iSNVs with FPR ≥ 0.1 as *Benign*. The reported *Damaging* category had true-positive rates (TPR) of 0.85 and 0.45 for on-ss and off-ss iSNVs, respectively, whereas TPRs for the *Possibly Damaging* category were 0.90 and 0.52 for on-ss and off-ss iSNVs (Fig. 3b, e).

### Evaluation of model performance using an independent test set

We further evaluated RegSNPs-intron performance with an independent dataset from ClinVar (described above). All of the ClinVar iSNVs that were also observed in HGMD or 1000 Genomes datasets were excluded from the training set to avoid overfitting. Consistent with the results described above, the RegSNPs-intron model showed better performance compared to SPANR. The RegSNPs-intron AUROCs were 0.96 and 0.95 for on-ss and off-ss iSNVs, respectively, whereas the SPANR AUROCs were 0.89 and 0.72 for on-ss and off-ss iSNVs, respectively (Fig. 3c, f). Similarly, RegSNPs-intron showed higher AUPRs. The RegSNPs-intron AUPRs were 0.92 and 0.66 for on-ss and off-ss iSNVs, whereas the SPANR AUPRs were 0.87 and 0.06, respectively (Additional file 1: Figure S7B and D). We did not include CADD in the comparison here since the ClinVar data were used in its original model training [17]. These results suggest that RegSNPs-intron exhibits stable performance and higher prediction accuracy compared to SPANR over different datasets.

### Allele frequency was inversely correlated with disease-causing probability

**Fig. 4:**
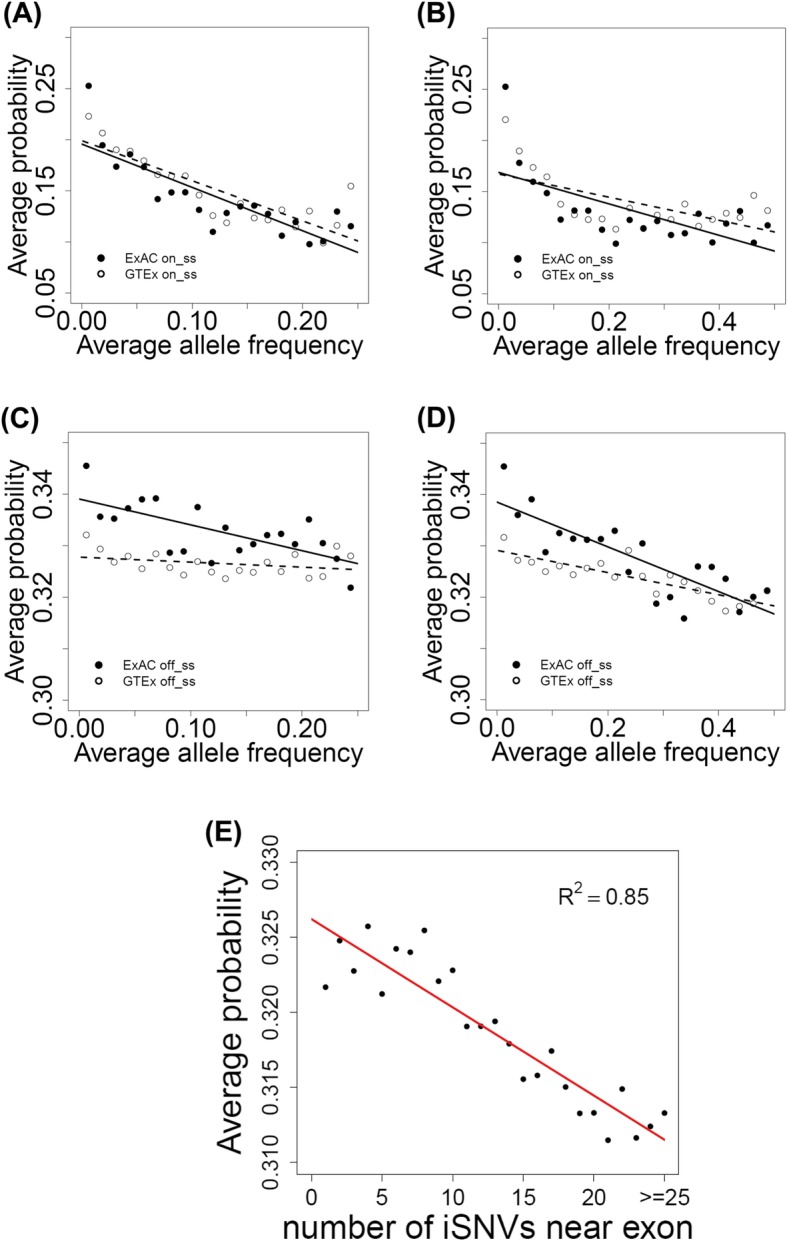
Correlations of disease-causing probability with allele frequency or number of nearby iSNVs for exon. **a**, **b** On-ss iSNVs. Correlation between disease-causing probability and allele frequency. Twenty bins were divided based on allele frequencies of the iSNVs collected from ExAC and GTEx. The *x*- and *y*-axes are the average allele frequency and predicted disease-causing probability for each bin. **a** Allele frequency scaled from 0.00 to 0.25. **b** Allele frequency scaled from 0.00 to 0.50. **c**, **d** Off-ss iSNVs, same as **a**, **b**. **e** Correlation between disease-causing probability and the number of exon-neighboring iSNVs. The *x*-axis is the number of exon-neighboring iSNVs (within 300 bp from the junction). Each value on the *y*-axis is the average (predicted) disease-causing probability of all exons having the number of neighboring iSNVs indicated by the corresponding value on the *x*-axis

Generally, allele frequency in the population should reflect the importance of the biological function of a variant [49–53]. Therefore, we examined the relationship between allele frequency and the predicted disease-causing probability of iSNVs obtained from the Exome Aggregation Consortium (ExAC) and the Genotype-Tissue Expression Project (GTEx), respectively. We focused on the low (0.0–0.25) and medium (0.0–0.50) scales of allele frequency, where the iSNVs were divided into 20 bins. For each bin, the average disease-causing probability for all iSNVs was calculated. Negative correlations were observed between allele frequency and disease-causing probability in either low or medium scale for both on-ss and off-ss iSNVs. Specifically, for the low scale, the correlations are as follows: ExAC *R* = − 0.832 and GTEx *R* = − 0.849 for on-ss iSNVs, and ExAC *R* = − 0.686 and GTEx *R* = − 0.313 for off-ss iSNVs (Fig. 4a, c). For the medium scale, the correlations are as follows: ExAC *R* = − 0.650 and GTEx *R* = − 0.603 for on-ss iSNVs, and ExAC *R* = − 0.834 and GTEx *R* = − 0.844 for off-ss iSNVs (Fig. 4b, d). This result is consistent with the findings of others that variants with higher disease-causing probability are less likely to occur in the general population [34]. Distributions of minor allele frequencies (MAF), in both the low and medium scales, for all iSNVs from ExAC and GTEx are provided in Additional file 1: Figure S9.

### Disease-causing iSNVs occur near exons associated with high disease-causing probability

We further evaluated the RegSNPs-intron predictions by investigating the functional importance of exons that are proximal to iSNVs. We hypothesized that functionally important exons do not tolerate nearby iSNVs. To test this hypothesis, we extracted 75,119 exons that had at least 1 iSNV within ± 300 bp of exon-intron boundaries based on ExAC data. One iSNV was randomly selected per exon, and the disease-causing probability was predicted using RegSNPs-intron. The probability values for all exons having the same number of neighboring (within ± 300 bp from junction) iSNVs were averaged. A significant negative correlation between the average disease-causing probability and the number of exon-neighboring iSNVs was observed (*R* = − 0.92, *p* value = 6.26 × 10^−11^) (Fig. 4e). The same analysis was also performed on 160,230 exons based on GTEx whole-genome sequencing data. A similar negative correlation was observed (*R* = − 0.78, *p* value = 0.07). This result indicates that pathogenic iSNVs tend to occur near functionally important exons.

### Prioritizing functional intronic variants associated with drug-induced cytotoxicity

We applied RegSNPs-intron to prioritize intronic variants that are associated with cellular sensitivity to clofarabine-induced cytotoxicity. We have previously performed genome-wide association studies (GWAS) for the clofarabine-response phenotype (AUC for drug-induced cytotoxicity curves) using 90 International HapMap lymphoblastoid cell lines (LCLs) of the CEU population [54]. SNVs moderately associated with clofarabine cytotoxicity (*p* value ≤ 0.05) were selected as seed markers. All 15,634 iSNVs that were in linkage disequilibrium (LD) with the seed markers and were located within ± 300 bp from the splice junction were used in the predictions. Among these candidate variants, 622 and 84 iSNVs were predicted to be *Damaging* (FPR ≤ 0.05) and *Possibly Damaging* (0.05 < FPR ≤ 0.1), respectively (706 in total), and 14,928 iSNVs were predicted to be *Benign* (FPR > 0.1).

### Experimental validation using ASSET-seq assay

**Fig. 5:**
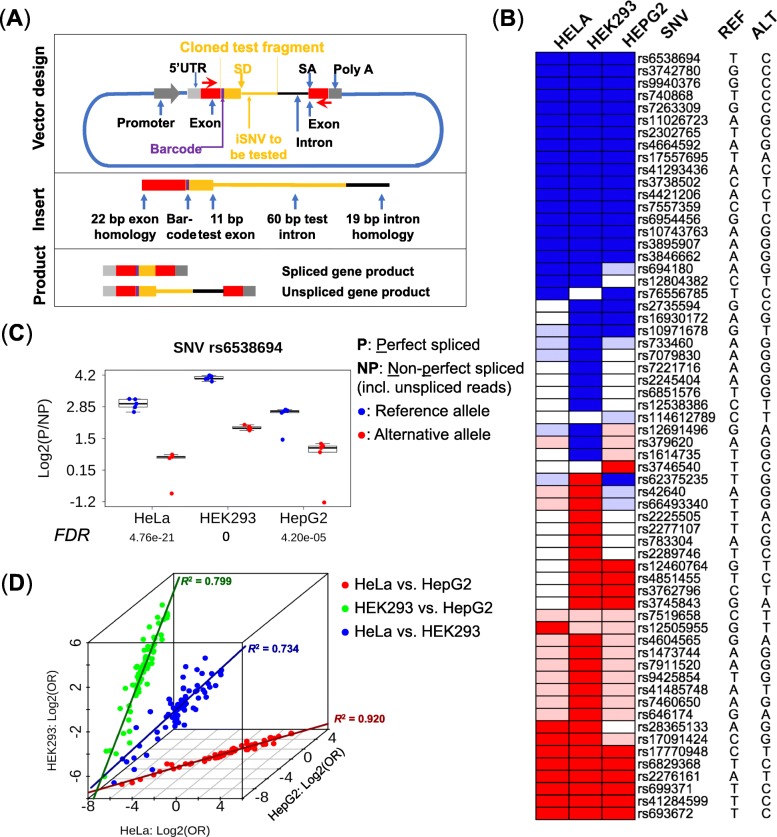
Experimental validation by ASSET-seq. **a** Plasmid design of the splicing assay. The insert oligo includes 11 bp of the proximal exon plus the first 60 bp of the intron containing the iSNV from individual genes (shown in orange), as well as universal plasmid exon (22 bp, red box) and intron (19 bp black line) homology segments for seamless insertion into the vector. RNA is transcribed in transfected cells from the 5′-UTR to the poly-A signal. PCR primers (red arrows) are used to amplify the RNA transcript. The assay produces spliced or unspliced transcripts (as well as other aberrant isoforms). Promoter, LTR RSV; SD, splice-donor site; SA, splice-acceptor site. **b** Summary of experimental results in three cell lines. Ref, reference allele; Alt, alternative allele. Blue color indicates an iSNV that induces a significant decrease in the spliced products (FDR ≤ 0.1); light blue indicates a decrease of spliced products that is not significant (FDR > 0.1). Red indicates a significant increase for spliced products whereas light red indicates an insignificant increase. Empty boxes are failed assays which were non-evaluable; iSNVs non-evaluable in all three cell lines (20 in total) were omitted. Validation rate (per cell line) is the percentage of assays showing a significant result in all evaluable assays. **c** Example of an iSNV-induced alteration of splicing outcome. The iSNV (*rs6538694*) suppressed the formation of the spliced reporter product, as consistently indicated in all three cell lines (*p* values ≤ 4.6 × 10^−5^). The *y*-axis is the log2 ratio of perfectly spliced reads (P) to aberrant (NP, non-perfect and unspliced) reads. *p* values were adjusted by FDR against all tested iSNVs. **d** Consistency of results in multiple cell lines. The *x*-axis is the log2 odds ratio of spliced and aberrant products (with respect to the reference and alternative alleles) for HeLa, and *y*- and *z*- axes are the log2 odds ratios for HepG2 and HEK293, respectively. Cell lines were compared pairwise for the iSNVs evaluable in both cell lines. Pearson’s correlations: HeLa versus HEK293 = 0.857 (blue dots, *p* value = 1.02 × 10^−22^), HeLa versus HepG2 = 0.959 (red dots, *p* value = 6.66 × 10^−40^), and HEK293 versus HepG2 = 0.894 (green dots, *p* value = 6.65 × 10^−29^). Solid lines in dark blue, red, and green mark the respective correlations

To experimentally validate the effects of the prioritized iSNVs on the splicing outcome, we designed a high-throughput functional reporter assay called ASSET-seq that entails inserting an oligo containing an iSNV into a modified Exontrap plasmid (Fig. 5a) [55]. The impact on splicing outcome of the tested iSNV was measured as the difference between the respective ratios of the sequencing reads supporting spliced and aberrant (including unspliced) transcripts for the reference and alternative alleles. The difference in the ratios for the two types of splicing outcomes between the reference and alternative alleles was analyzed by a mixed-effect model (see the “Methods” section).

We used ASSET-seq to test the effects of 82 iSNVs on splicing outcomes in 3 different human cell lines: HeLa, HEK293, and HepG2. These cell lines, which originate from three different tissue types, are commonly used in research studies and were used to demonstrate the variability in splicing activity among different tissues. Additionally, they also exemplify the technical reproducibility of the assay, as well as enable future studies linking splicing analysis with molecular biological outcomes. The 82 test variants were chosen from the 706 prioritized RegSNPs-intron candidates (FPR ≤ 0.1) as being located in the intron on the 3′-side of the test exon and within 60 nt from the exon-intron junction, based on assay design requirements (see details in the “Methods” section). Upon removing 20 failed assays (e.g., resulting from transfection or PCR failure), the percentages of the 62 remaining iSNVs showing significant splicing impact, i.e., validation rates, for the 3 cell lines were HeLa 64.4%, HEK293 96.6%, and HepG2 64.7% (FDR ≤ 0.1 in accord with the FPR; Fig. 5b and Additional file 3: Table S2). One specific example, *rs*6538694 in the *HAL* gene, is shown in Fig. 5c. Across all three cell lines (five replicates each), a significantly higher percentage of spliced gene products was observed for the reference allele compared to the alternative allele (*p* values ≤ 4.6 × 10^−5^). For the reference allele, the average percentage of sequencing reads supporting the spliced gene product was 88.4%, while this percentage dropped to 57.1% for the alternative allele. Moreover, we observed high consistency for the impacts of individual iSNVs on splicing outcomes across the multiple cell lines (Fig. 5d).

## Discussion

The major conclusion of the current study is that RNA splicing, protein structural, and evolutionary conservation features all contribute to iSNV pathogenicity characterization. By integrating these three feature categories, the RegSNPs-intron algorithm efficiently evaluates the disease-causing probabilities of iSNVs in silico. These conclusions are based on the following evidence. First, we demonstrated that disease-causing iSNVs affect alternative splicing, localize to conserved genomic regions, and are associated with functional domain-encoding exons. Next, we provided strong evidence that RegSNPs-intron has superior accuracy in computing the disease-causing probabilities for iSNVs compared to existing tools such as SPANR and CADD, based on 1000 Genomes and HGMD data, as well as with independent ClinVar data. Furthermore, we applied RegSNPs-intron to a GWAS dataset of drug cytotoxicity and experimentally validated the impact of prioritized iSNVs on splicing via ASSET-seq. Taken together, our findings strongly support the overall concept that the RegSNPs-intron algorithm, combined with the ASSET-seq assay, will facilitate studies on the regulatory functions of iSNVs and their potential roles in disease and/or drug response.

Although information on variant-induced disruption of splicing and variant conservation has been used to evaluate the impact of synonymous variants [32], our previous studies have shown that protein structural features greatly improve the prioritization of pathogenic micro-insertions/deletions as well as alternative-splicing events [34, 35]. Prompted by our earlier findings, we proposed that protein structural features might also be informative in predicting the pathogenic effects of iSNVs. This idea was also supported by the finding that pathogenic iSNVs tend to be localized in the vicinity of exons encoding functionally important protein domains. Following the common practice of integrating multi-level features, as in algorithms such as SPANR and CADD, RegSNPs-intron constitutes the first bioinformatics tool specifically designed to predict pathogenic iSNVs.

The improved accuracy of RegSNPs-intron compared with SPANR and CADD in predicting pathogenic iSNVs may also be attributed to the differences in the training and testing datasets used in the various algorithms. For instance, the goal of SPANR is to evaluate the impact of variants on splicing outcome, regardless of whether such changes are pathogenic. Therefore, the selections of the training data were based on different criteria. To this end, RegSNPs-intron focuses on the pathogenic impact of iSNVs by selecting specific training dataset and molecular features. In addition, to avoid potential bias on model performance resulting from differences in the selected datasets, the accuracy of RegSNPs-intron was also evaluated on an independent dataset ClinVar.

Intronic variants are typically identified by whole-genome sequencing (WGS), but they can also be captured by whole-exome sequencing (WES), particularly those iSNVs close to splice junctions that may be functionally important. To estimate the number of intronic variants identified in WES, we surveyed all the genetic variants documented in the ExAC database [56], which includes high-quality exome sequencing data from 60,706 unrelated individuals from a variety of large-scale sequencing projects such as the NHLBI exome sequencing project (ESP) and the 1000 Genomes project. Among 7,908,659 documented SNVs in the ExAC database, these iSNVs account for 52.2% (4,126,724); therefore, identifying which of these iSNVs are important for disease pathogenesis is crucial for advancing human genetics research. Thus, the RegSNPs-intron algorithm should serve as a valuable tool for the prioritization of intronic variants detected through WES and WGS for functional analysis.

In order to experimentally validate the impact of predicted pathogenic iSNVs on splicing regulation, we developed an innovative experimental approach, ASSET-seq. Although we tested a relatively small number of variants in this study, ASSET-seq can operate on a much larger scale. However, despite the effectiveness of this high-throughput assay, there are a few limitations. First, ASSET-seq can only validate the potential roles of candidate iSNVs on splicing; it was not designed to test their pathogenic roles. As a result, the impact that mis-spliced exons could have on protein structure was not evaluated, which is a potential source for false-positive results. For example, if an iSNV was to mis-regulate splicing of a functionally neutral exon, it would appear to be positive in ASSET-seq. Additional experimental assays are required to examine the impact of such iSNVs on specific phenotypes. Secondly, our results were influenced by technical noise from multiple sources, such as cell transfection, sequencing, or PCR, that served to confound the analysis. Since these effects could not be entirely prevented, we considered the heterogeneity among the sample replicates and applied the generalized mixed-effect model to characterize the significance of change in splicing outcomes (i.e., correlation between allele types and spliced products). Interestingly, most of the RegSNPs-intron-predicted candidates displayed significant impact on splicing regulation in multiple cell lines. Specifically, HEK293 cells exhibited an excellent validation rate and low noise, whereas HeLa and HepG2 cells had lower validation rates, possibly due to larger data variabilities (higher noise). Thus, our validation results confirm the effectiveness of RegSNPs-intron in prioritizing iSNVs.

## Conclusions

Our results show that integrating RNA splicing, protein structural, and evolutionary conservation features leads to superior characterization of disease-causing iSNVs. Using RegSNPs-intron and ASSET-seq in tandem enables the effective prioritization of disease-causing iSNVs. This is expected to accelerate the identification of pathogenic iSNVs, a core task of genome-wide sequencing studies.

## Methods

### Splicing features

A junction score for the closest exon boundary of each iSNV was calculated based on the position weight matrices (PWMs) derived from canonical splice sites [42]. The junction score was measured by summing the information contents of positions from − 3 to + 7 for donor sites, and positions from − 13 to + 1 for acceptor sites. In addition, for on-ss iSNVs, the change in junction score caused by allele substitution was also computed and used as a feature.

The impact of iSNVs on RBP binding affinity was measured based on a total of 201 PWMs (position weight matrices) obtained from the RBPDB and cisBP-RNA databases [57, 58]. For each iSNV, we compute the following two measures to quantify the impact on the binding of a specific RBP:

i) The magnitude (***M*** value) of RBP binding change upon each iSNV, which was calculated as the logarithmic ratio between the custom-defined RBP binding scores with respect to the alternative allele and the reference allele.
ii) An estimated probability (***P*** value) of RBP binding event switch upon each iSNV, i.e., an RBP binding site switches to a non-binding site or vice versa given the two respective alleles.

Denote *p* as an RBP with a binding motif with length *k* and *q* = {*q*_*i*_ = *A*, *T*, *C*, *G*, *i* = 1…*k*} as a nucleotide sequence with length *k*. As a prerequisite for calculating the ***M*** and ***P*** values from the PWM data, we first calculated the matching score of the sequence *q* to the PWM of *p* as:

$$ {S}_q^p={\sum}_{i=1}^k{s}_{i,{q}_i}^p $$

$$ {s}_{i,j}^p={\log}_2\frac{\left({n}_{i,j}^p+{c}_{i,j}\right)/\left(N+{\sum}_{j\in \left\{A,C,G,T\right\}}{c}_{i,j}\right)}{d_j},i=1,\dots, k,j=A,T,C,G $$

where ${s}_{i,j}^p$ is the logarithmic ratio of the observed frequency of a specific nucleotide *j* in the *i*th position of the PWM of *p* versus the random background frequency, ${n}_{i,j}^p$ is the count of base *j* = *A*, *T*, *C*, *G* on the *i*th position in the PWM of *p*, and *c*_*i*, *j*_ is a pseudo-count to avoid the negative infinite value of ${s}_{i,j}^p$ when ${n}_{i,j}^p=0.$ *N* is the total number of binding sites used to derive the PWM, and *d*_*j*_ is the prior frequency of base *j*. In this study, we set *c*_*i*, *j*_ as $\sqrt{N}$ and assumed a constant *d*_*j*_ = 0.25 for *j* = *A*, *T*, *C*, *G.*

To evaluate if a certain iSNV may significantly impact the binding event for an RBP binding site, we first estimate two empirical distributions—(1) the distribution of ${S}_q^p$ for the nucleotide sequences that are true RBP binding site of *p*, and (2) the distribution of ${S}_q^p$ for the nucleotide sequences that are not RBP binding site of *p*.

As demonstrated in previous studies [33, 36], it is rational to assume the empirical distributions of the matching scores ${S}_q^p$ follows a Gaussian distribution. Specifically, mean and variance of ${S}_q^p$ can be estimated by *M*_*p*_ and *V*_*p*_ defined below:

$$ {M}_p={\sum}_{i=1}^k\sum \limits_{j\in \left\{A,C,G,T\right\}}{f}_{i,j}^p\times {s}_{i,j}^p $$

$$ {V}_p={\sum}_{i=1}^k\sum \limits_{j\in \left\{A,C,G,T\right\}}\left({f}_{i,j}^p\times {s_{i,j}^p}^2-{\left({f}_{i,j}^p\times {s}_{i,j}^p\right)}^2\right) $$

where ${f}_{i,j}^p$ is the frequency of base *j* = *A*, *T*, *C*, *G* at the *i*th position of the RBP binding site *p*. With the PWM of *p*, for the true binding site, ${f}_{i,j}^p\triangleq \frac{2^{s_{i,j}^p}}{4}=\frac{n_{i,j}^p+{c}_{i,j}}{N+{\sum}_{j\in \left\{A,C,G,T\right\}}{c}_{i,j}}$. To estimate the mean and variance of ${S}_q^p$ for the sequences that do not form an RBP binding site, we assume ${f}_{i,j}^p=0.25$ for *j* = *A*, *T*, *C*, *G* at any position *i*, i.e., an even distribution of the background. The rationale of this assumption is that only a very small number of specific sequences may form the binding site; hence, in their complement set, the frequency of each base at each position tends to be even.

With these assumptions, the two empirical distributions of ${S}_q^p$ for the nucleotide sequences that serve as true RBP binding site or non-RBP binding sites of *p* can be computed. It is noteworthy the two Gaussian distribution of the matching scores for binding (*B*) and non-binding (*NB*) sites always exhibit different means and variations, (as illustrated in Additional file 1: Figure S10). With the two empirical distribution computed, we further computed the ***M*** value—the magnitude of how an iSNV affects RBP binding, as detailed below:

Denote *A* as a sequence with the alternative allele of the iSNV, and *R* as the sequence with the reference allele, their matching scores to the PWM of RBP binding site *p* were first computed and denoted as ${S}_A^p$ and ${S}_R^p$. We developed a custom score, denoted Ω(*S*) = Φ(*S*, *B*)/(1 − Φ(*S*, *NB*)), where *S* = ${S}_A^p$ or ${S}_R^p$. Ω is the ratio of a Φ score for a binding event against the one for a non-binding event, given the matching score *S* of a specific allele. Here, Φ(*S*, *B*) or Φ(*S*, *NB*) is the cumulative distribution function (CDF) of the Gaussian distribution characterized by *M*_*p*_ and *V*_*p*_ (described earlier) for any PWM matching score *S* (Additional file 1: Figure S10):

$$ \Phi \left(S,B\right)={\int}_{-\infty}^{\mathrm{S}}\frac{1}{\sqrt{2\pi {V}_p(B)}}{e}^{-\frac{1}{2}{\left(\frac{x-{M}_p(B)}{\sqrt{V_p(B)}}\right)}^2}d(x) $$

$$ \Phi \left(S, NB\right)={\int}_{-\infty}^{\mathrm{S}}\frac{1}{\sqrt{2\pi {V}_p(NB)}}{e}^{-\frac{1}{2}{\left(\frac{x-{M}_p(NB)}{\sqrt{V_p(NB)}}\right)}^2}d(x) $$

where *M*_*p*_(*B*) (*M*_*p*_(*NB*)) and *V*_*p*_(*B*) (*V*_*p*_(*NB*)) are the mean and variance for binding (non-binding) events.

As shown in Additional file 1: Figure S10, Φ(*S*, *B*) is the red-shaded area under the density curve of binding events, symbolizing how likely to observe a particular *S* given that the sequence is a RBP binding site. On the other hand, 1 − Φ(*S*, *NB*) is the blue-shaded area under the density curve of non-binding events, indicating how likely to observe the *S* if the sequence is a non-binding site. They are designed in this way because we assume that it is more likely to observe a larger matching score to the PWM of an RBP if the specific allele promotes RBP binding, and in contrast, it should be less probable to observe a large matching score if the allele disrupts RBP binding [36].

Thus intuitively, Ω(*S*), the ratio between Φ(*S*, *B*) and 1 − Φ(*S*, *NB*), is the relative extent of how likely the sequence is a binding site compared to a non-binding site, given a specific allele (*S* = ${S}_A^p$ or ${S}_R^p$). Then, we defined:

$$ \boldsymbol{M}={\log}_2\frac{\Omega \left({S}_A^p\right)}{\Omega \left({S}_R^p\right)} $$

for each RBP (*p*).

The probability (***P*** value) that a locus switches between RBP binding and non-binding with and without the variant can be defined as the sum of two probabilities in which the different alleles correspond to binding (*B*) and non-binding (*NB*) events given the observed matching scores ${S}_A^p$ and ${S}_R^p$. For each specific RBP binding site *p*, we simplifiy the denotation as ${S}_A^p\triangleq {S}_A$ and ${S}_R^p\triangleq {S}_R$ in all the formulas following below:

$$ \boldsymbol{P}\left(\mathrm{Switch}\right)=P\left(R=B,A= NB|{S}_R,{S}_A\right)+P\left(\mathrm{R}= NB,A=B|{S}_R,{S}_A\right) $$

Here, *R* (or *A*) = *B* means the reference (or alternative) allele corresponds to an RBP binding event, and *R* (or *A*) = *NB* indicates it is a non-binding event. We assume that the allele identity of the sequence (*R* and *A*) is independent of each other, and observations of the matching scores (*S*_*R*_ and *S*_*A*_) are also independent. Therefore, by the Bayes law, the above probability can be transformed into the following cascade of equations:

$$ {\displaystyle \begin{array}{c}P\left(\mathrm{Switch}\right)=P\left(R=B,A= NB|{S}_R,{S}_A\right)+P\left(R= NB,A=B|{S}_R,{S}_A\right)\\ {}=\frac{P\left(R=B,A= NB\right)P\left({S}_R,{S}_A|R=B,A= NB\right)}{P\left({S}_R,{S}_A\right)}+\frac{P\left(R= NB,A=B\right)P\left({S}_R,{S}_A|R= NB,A=B\right)}{P\left({S}_R,{S}_A\right)}\\ {}=\frac{P(B)P(NB)P\left({S}_R|B\right)P\left({S}_A| NB\right)}{P\left({S}_R\right)P\left({S}_A\right)}+\frac{P(NB)P(B)P\left({S}_R| NB\right)P\left({S}_A|B\right)}{P\left({S}_R\right)P\left({S}_R\right)}\\ {}=\frac{P(B)P(NB)\left[P\left({S}_R\Big\Vert B\right)P\left({S}_A| NB\right)+P\left({S}_R| NB\right)P\left({S}_A|B\right)\right]}{\left(P(B)P\left({S}_R|B\right)+P(NB)P\left({S}_R| NB\right)\right)\left(P(B)P\left({S}_A|B\right)+P(NB)P\left({S}_A| NB\right)\right)}\\ {}=\frac{P(B)\left(1-P(B)\right)\left[P\left({S}_R|B\right)P\left({S}_A| NB\right)+P\left({S}_R| NB\right)P\left({S}_A|B\right)\right]}{\left(P(B)P\left({S}_R|B\right)+\left(1-P(B)\right)P\left({S}_R| NB\right)\right)\left(P(B)P\left({S}_A|B\right)+\left(1-P(B)\right)P\left({S}_A| NB\right)\right)}\end{array}} $$

given that *P*(*NB*) = 1 − *P*(*B*) for any sequence.

Since *S*_*R*_ and *S*_*A*_ follow Gaussian distribution for both *B* and *NB* (Additional file 1: Figure S10), the following equalities are established:

$$ P\left({S}_R|B\right)=\Phi \left({S}_R,B\right) $$

$$ P\left({S}_R| NB\right)=\Phi \left({S}_R, NB\right) $$

$$ P\left({S}_A|B\right)=\Phi \left({S}_A,B\right) $$

$$ P\left({S}_A| NB\right)=\Phi \left({S}_A, NB\right) $$

and each Φ value is calculated as described earlier.

Here, *P*(*B*) is a prior probability that a sequence is an RBP binding site. Since it is unknown, we denote it as *x* ∈ [0, 1] and assume *x* follows a beta distribution. Shape parameters *α* and *β* for the beta distribution were specific to each sequence and estimated by enforcing the following constrains: (1) *α*, *β* > 1; (2) mode of the beta distribution (mode = (*α* − 1)/(*α* + *β* − 2)) equals to 1/2^IC^, where IC is the information content (i.e., sum of the expected self-information of the elements) of the PWM; and (3) the beta cumulative distribution function at 1/10th of the mode (i.e., mode/10) equals a pre-defined level 0.005.

Given any value of *x*, the ***P***(Switch) becomes the conditional probability with respect to the given *x*:

$$ \boldsymbol{P}\left(\mathrm{Switch}\ \right|\ x\Big)=\frac{x\left(1-x\right)\left[\Phi \left({S}_R,B\right)\Phi \left({S}_A, NB\right)+\Phi \left({S}_R, NB\right)\Phi \left({S}_A,B\right)\right]}{\left(x\Phi \left({S}_R,B\right)+\left(1-x\right)\Phi \left({S}_R, NB\right)\right)\left(x\Phi \left({S}_A,B\right)+\left(1-x\right)\Phi \left({S}_A, NB\right)\right)} $$

with all calculated Φ values substituted in.

According to the Bayesian theorem, the probability ***P***(Switch) can be calculated by the following integral:

$$ \boldsymbol{P}\left(\mathrm{Switch}\right)={\int}_0^1\boldsymbol{P}\left(\mathrm{Switch}\ \right|\ x\Big)\bullet {f}_{\mathrm{beta}}\left(x,\alpha, \beta \right) dx $$

where *f*_beta_ is the probability density function of beta distribution, with *α* and *β* estimated as described above.

Based on all above calculations, the two measures ***M*** and ***P*** were derived for each of the 201 RBPs and used as the splicing features.

### Protein structural features

For each iSNV, the protein structural features of its closest exons were evaluated. The protein-disorder score, secondary structure, and solvent accessible surface area (ASA) were precomputed for all known protein-coding genes using SPINE-D and SPINE-X [43, 44]. The known protein domains were extracted from the Pfam database [45]. Percentages of the closest exon regions that overlap with Pfam domains were measured. The post-translational modification sites (PTMs) were extracted from the dbPTM 3.0 database [46]. The number of PTM sites per 100 amino acids encoded by the closest exons was also calculated.

### Evolutionary conservation features

Base-wise conservation scores (PhyloP) of 99 vertebrate genomes were downloaded from UCSC Genome Browser [59]. The scores on the iSNVs loci, as well as the average scores of the 3-bp and 7-bp window regions around the iSNVs, were extracted and used in machine learning.

### Machine learning model

Separate random forest classifiers were built for on-ss and off-ss iSNVs, respectively. A grid search strategy with threefold cross-validation was used on the training set to fine-tune the hyperparameters, such as the number of trees and the maximum tree depth. The source code can be accessed by public repository or DOI reference links [60].

### ClinVar database iSNVs

ClinVar (version 2016/05/31) was downloaded from NCBI (ftp://ftp.ncbi.nlm.nih.gov/pub/clinvar/). We extracted the SNVs located in intronic regions. To ensure the quality of data, we only included the iSNVs that were confirmed by at least two submitters, with the exception of pathogenic off-ss iSNVs where we only required a single submitter due to the limited number of such iSNVs.

### GTEx and ExAC database iSNVs

SNVs from whole-genome sequencing data in GTEx release v6 were downloaded in the VCF format. We focused on the iSNVs within 300 bp of exon-intron boundaries. In total, there were 17,194 on-ss iSNVs and 630,557 off-ss iSNVs. Similarly, we also downloaded the variants from whole-exome sequencing data in ExAC r0.3.1, where there were 368,489 on-ss iSNVs and 2,314,839 off-ss iSNVs. The corresponding allele frequencies were calculated for correlation analysis with predicted disease-causing probabilities.

### ASSET-seq plasmid construction

The modified Exontrap plasmid is shown in Fig. 5a, and the sequence is provided in Additional file 4: Text S1. The test oligos consisted of 11 bp of the upstream exon and 60 bp of the adjacent intron containing the iSNV to be tested. Additional 22-bp exonic and 19-bp intronic sequences homologous to the vector were also included for the seamless insertion of the oligos into the plasmid body. Further, a single nucleotide barcode was introduced to indicate whether the transcript came from the wild-type or variant construct. The 113-bp oligos containing different test iSNVs were synthesized in parallel as a pool using OligoMix (LC Sciences, Houston, TX). In the present study, the ASSET-seq assays contained 82 pairs of reference and variant test sequences. The synthesized oligos were then cleaved from the chip and amplified via high-fidelity PCR with primers paired to the exon and intron homology sequences (Additional file 4: Text S1). The pooled oligos were directionally inserted into the Exontrap plasmid using the NEBuilder HiFi DNA Assembly Reaction (New England Biolabs, Ipswich MA). The assembled plasmids were transformed into bacteria and plated on LB agar plates containing ampicillin. The resulting colonies were scraped and grown in LB + ampicillin medium. Plasmid DNA was isolated using HiSpeed Plasmid Maxi kit (Qiagen, Germantown, MD).

### Transfection of cell culture

The plasmid library was used to transfect three human cell lines: HeLa, HEK293, and HepG2. The cells were seeded at a density of 0.9 × 10^5^ in 24-well plates. Each plate contained 5 biological replicates per cell line. Twenty-four hours after cell plating, 500 ng of the library pool was complexed with 1.5 μL of Lipofectamine 3000 Reagent (Thermo Fisher Scientific, Waltham, MA) in 50 μL of Opti-MEM media as per the manufacturer’s instructions before adding the transfection mixture to each well. Cell culture and transfection reagents were used without antibiotics. HeLa, HEK293, and HepG2 were authenticated using IDEXX Bioanalytics’ CellCheck 9 Plus (Columbia, MO). All were found to be within IDEXX’s range of positive identity matching.

### RNA isolation and cDNA synthesis

Transfected cells were lysed in situ 48 h after transfection, and total RNA was isolated using miRNeasy mini kit with the optional DNase digestion step (Qiagen, Germantown MD) following the manufacturer’s protocol. Using 285–400 ng RNA, cDNA was synthesized with QuantiTect Reverse Transcription kit (Qiagen, Germantown, MD) following the manufacturer’s protocol.

### Molecular barcoding

To identify the source cell line and replicate of the RNA transcripts, cDNA generated from the plasmid library in the transfected cells was PCR amplified using barcoded primers. A unique 6-nt sequence was added to the 5′-end of the forward and reverse primer for identification (Additional file 4: Text S1). In separate PCR reactions, 2 μL cDNA were amplified in a 50-μL volume containing 2× Invitrogen Platinum SuperFi PCR Master Mix (Thermo Fisher Scientific, Waltham, MA) using 1 μM (final concentration) barcoded primers. PCR conditions used were 98 °C for 30 s, 66.6 °C for 5 s, and 72 °C for 15 s for 28 cycles. PCR samples were purified using the MinElute PCR Purification kit (Qiagen, Germantown, MA) following the manufacturer’s protocol and quantified with Qubit dsDNA BR Assay kit (Thermo Fisher Scientific, Waltham, MA). For sequencing by the NextSeq 500 platform (Illumina, San Diego, CA), 150 ng of each sample was pooled. Pooled samples also contained an equal amount of the original plasmid library with identification barcodes added by PCR as above. Pooled samples contained 20 uniquely barcoded sequence groups representing 5 biological replicates for 3 cell lines plus 5 input plasmid libraries.

### Next-generation sequencing

The pooled PCR products were sequenced using the NextSeq 500 platform (Illumina, Inc., San Diego, CA). The sequencing library was created by end-polishing the barcoded PCR products, followed by adapter ligation and amplification. The resulting library was quantified, and its quality was assessed with the Agilent Bioanalyzer (Agilent Technologies, Santa Clara, CA). Approximately 90 million usable reads were generated. Raw reads were generated as fastq files for bioinformatics analysis.

### Bioinformatics analysis for sequencing data

Illumina sequencing adapters were first removed from the raw reads in the fastq files using the tool *cutadapt* (v1.9.1) [61]. Then, the reads were demultiplexed into the 20 sequence groups according to the barcodes. Sequencing reads were aligned to the transcripts using STAR (Spliced Transcripts Alignment to a Reference, v2.5.3a) [62]. The reference sequence for the alignment was built based on the plasmid (Additional file 4: Text S1), and read counts for the spliced and aberrant (including unspliced) transcripts were documented.

### Statistical analysis

Assuming heterogeneity among the sample replicates, we applied the generalized linear mixed-effect model to characterize the difference of splicing patterns between reference and alternative alleles in each of the three experimental cell lines. The splicing outcome was described by the counts of sequencing reads supporting the spliced and aberrant transcripts. Assuming *Y* is the sequencing read count following negative binomial distribution and *x*_allele_ and *x*_splice_ are two binary variables (i.e., 0 or 1), we set up the following regression equation with respect to the (logarithm of) expectation *E*(*Y*):

$$ \log \left(E(Y)\right)={\beta}_0+{\beta}_1\bullet {x}_{\mathrm{allele}}+{\beta}_2\bullet {x}_{\mathrm{splice}}+{\beta}_3\bullet {x}_{\mathrm{allele}}:{x}_{\mathrm{splice}}+b\bullet {\varepsilon}_{\mathrm{replicate}} $$

$$ {x}_{\mathrm{allele}}\in \left\{0-\mathrm{ref},1-\mathrm{alt}\right\},{x}_{\mathrm{splice}}\in \left\{0-\mathrm{spliced},1-\mathrm{aberrant}\right\} $$

Here, *ε* is the random effect among the multiple replicates. The *p* value of coefficient *β*_3_ determines whether there is a significant change in splicing outcome between the two alleles, i.e., *x*_splice_ significantly correlates to *x*_allele_.

## Supplementary information

**Additional file 1: Figure S1.** Detailed technical protocol. **Figure S2.** Data pre-processing. **Figure S3.** Distribution of changes in splice-junction scores. **Figure S4.** Average RBP binding score changes. **Figure S5.** Cumulative probability density of protein structural features. **Figure S6.** Quantile-quantile plot of PhyloP conservation scores. **Figure S7.** Model evaluation in terms of precision-recall curves. **Figure S8.** Performance of sub-models with features from each individual category. **Figure S9.** Distribution of minor allele frequencies of ExAC and GTEx iSNVs. **Figure S10.** Demonstration of PWM-derived matching score distribution.

**Additional file 2: Table S1.** Complete list of model features and their individual predictive powers.

**Additional file 3: Table S2.** Additional data for ASSET-seq experiment result.

**Additional file 4: Text S1.** Sequence of the modified Exontrap plasmid in ASSET-seq.

**Additional file 5.** Review history.

## Data Availability

Raw sequencing data of ASSET-seq have been deposited in NCBI’s Gene Expression Omnibus (GEO) and are accessible through GEO Series accession number GSE138130 (https://www.ncbi.nlm.nih.gov/geo/query/acc.cgi?acc=GSE138130) [63]. All the other data generated or analyzed during this study are included in this published article and the supplementary information files. RegSNPs-intron is available at https://regsnps-intron.ccbb.iupui.edu, with source code deposited at Github (https://github.com/yunliu/regsnp_intron) [60] and released under the MIT license.
