# Supplementary material for: RegSNPs-intron: a computational framework for predicting pathogenic impact of intronic single nucleotide variants
Source: Genome Biol. 2019 Nov 28;20:254. doi: 10.1186/s13059-019-1847-4 (PMC6883696; doi:10.1186/s13059-019-1847-4)
Supplement: Supplementary file 1 — Additional file 1: Figure S1. Detailed technical protocol. Figure S2. Data pre-processing. Figure S3. Distribution of changes in splice-junction scores. Figure S4. Average RBP binding score changes. Figure S5. Cumulative probability density of protein structural features. Figure S6. Quantile-quantile plot of PhyloP conservation scores. Figure S7. Model evaluation in terms of precision-recall curves. Figure S8. Performance of sub-models with features from each individual category. Figure S9. Distribution of minor allele frequencies of ExAC and GTEx iSNVs. Figure S10. Demonstration of PWM-derived matching score distribution. [file 13059_2019_1847_MOESM1_ESM.pdf]

Fig S1.

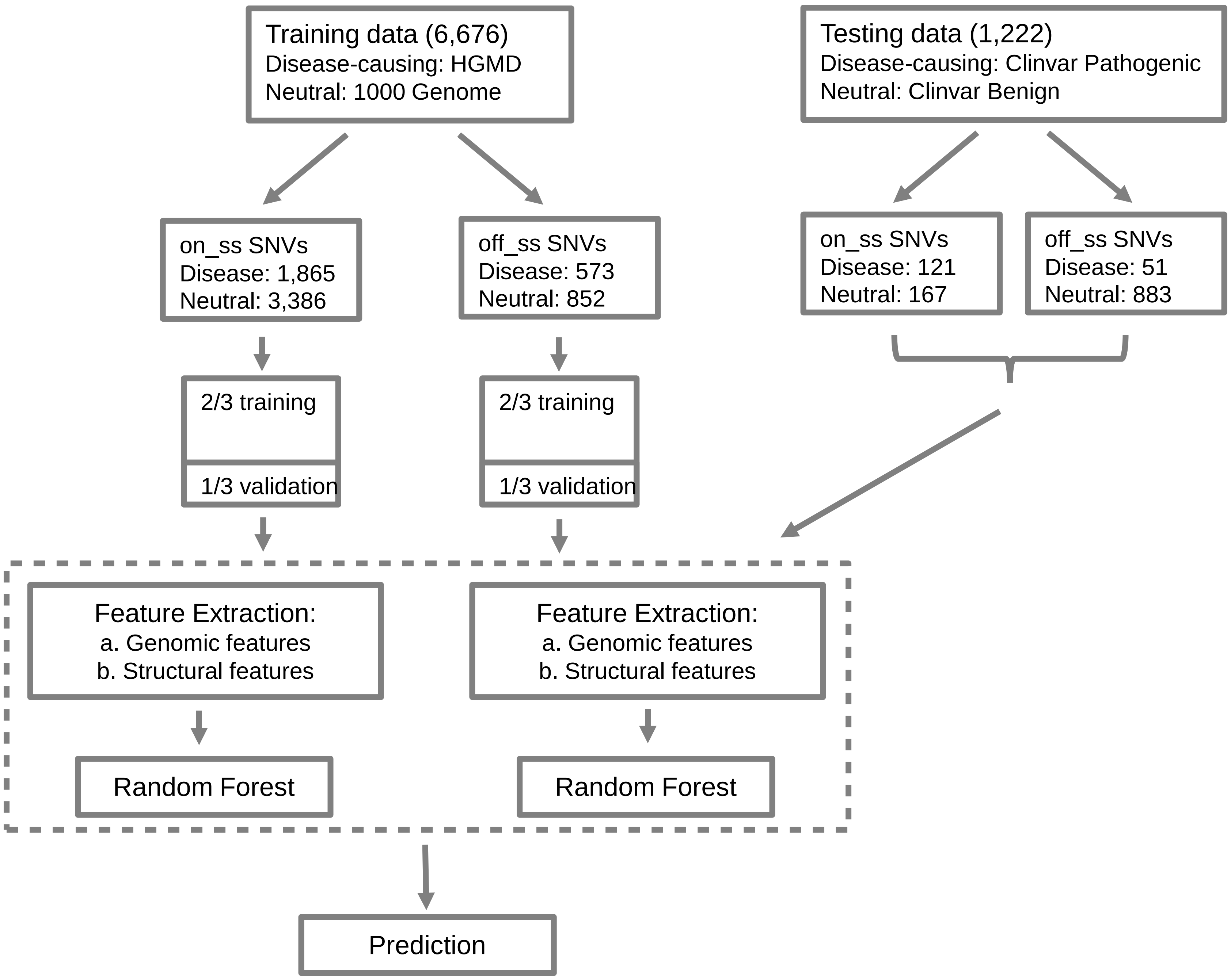
Training data (6,676)
Disease-causing: HGMD
Neutral: 1000 Genome
Testing data (1,222)
Disease-causing: Clinvar Pathogenic
Neutral: Clinvar Benign
on_ss SNVs
Disease: 1,865
Neutral: 3,386
off_ss SNVs
Disease: 573
Neutral: 852
on_ss SNVs
Disease: 121
Neutral: 167
off_ss SNVs
Disease: 51
Neutral: 883
2/3 training
1/3 validation
2/3 training
1/3 validation
Feature Extraction:
a. Genomic features
b. Structural features
Feature Extraction:
a. Genomic features
b. Structural features
Random Forest
Random Forest
Prediction

Fig S2.

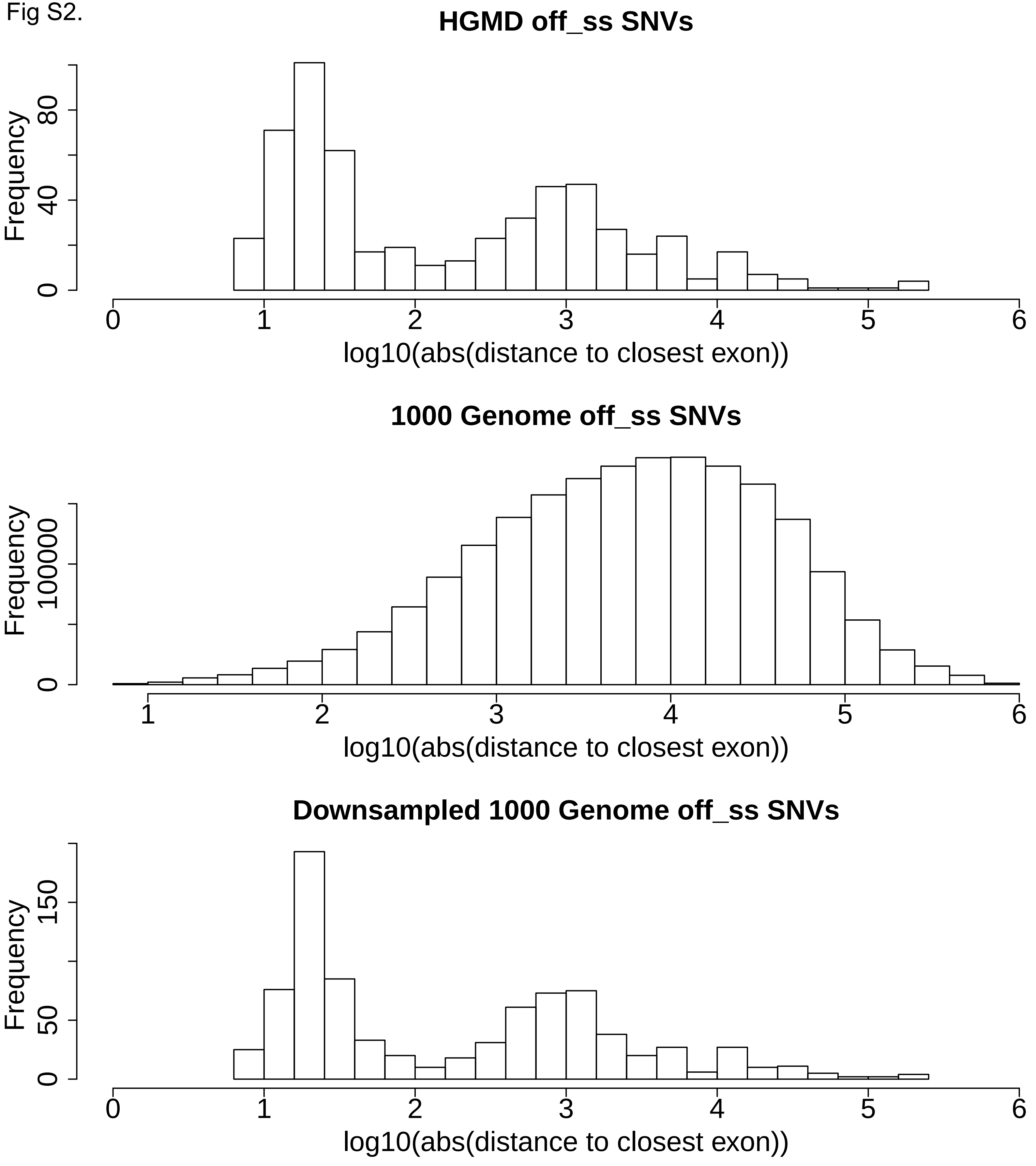
HGMD off_ss SNVs
Frequency
0
40
80
0 1 2 3 4 5 6
log10(abs(distance to closest exon))
1000 Genome off_ss SNVs
Frequency
0
100000
1 2 3 4 5 6
log10(abs(distance to closest exon))
Downsampled 1000 Genome off_ss SNVs
Frequency
0
50
150
0 1 2 3 4 5 6
log10(abs(distance to closest exon))

Fig S3.

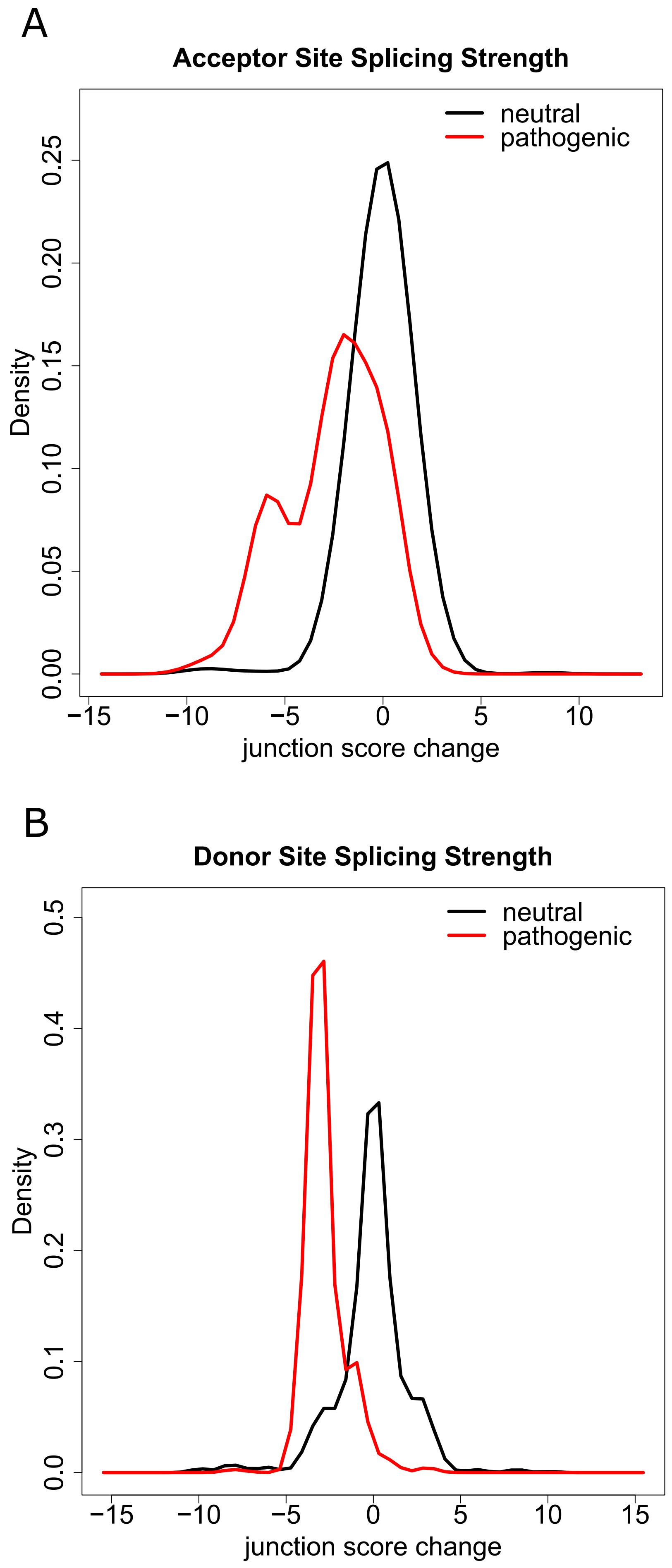
A
Acceptor Site Splicing Strength
neutral
pathogenic
Density
0.00
0.05
0.10
0.15
0.20
0.25
−15
−10
−5
0
5
10
junction score change
B
Donor Site Splicing Strength
neutral
pathogenic
Density
0.0
0.1
0.2
0.3
0.4
0.5
−15
−10
−5
0
5
10
15
junction score change

Fig S4.

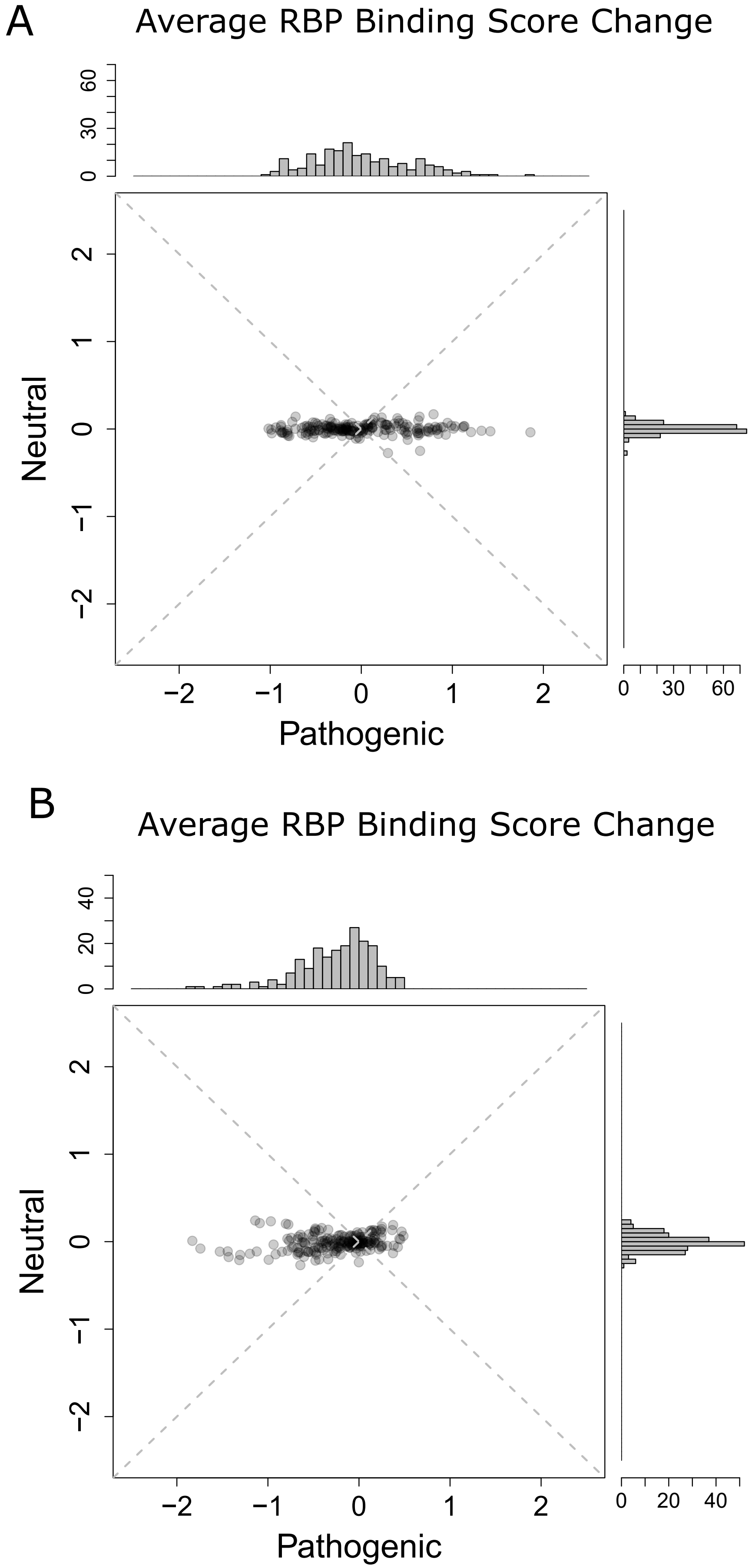

A
Average RBP Binding Score Change
Neutral
Pathogenic
B
Average RBP Binding Score Change
Neutral
Pathogenic

Fig S5.

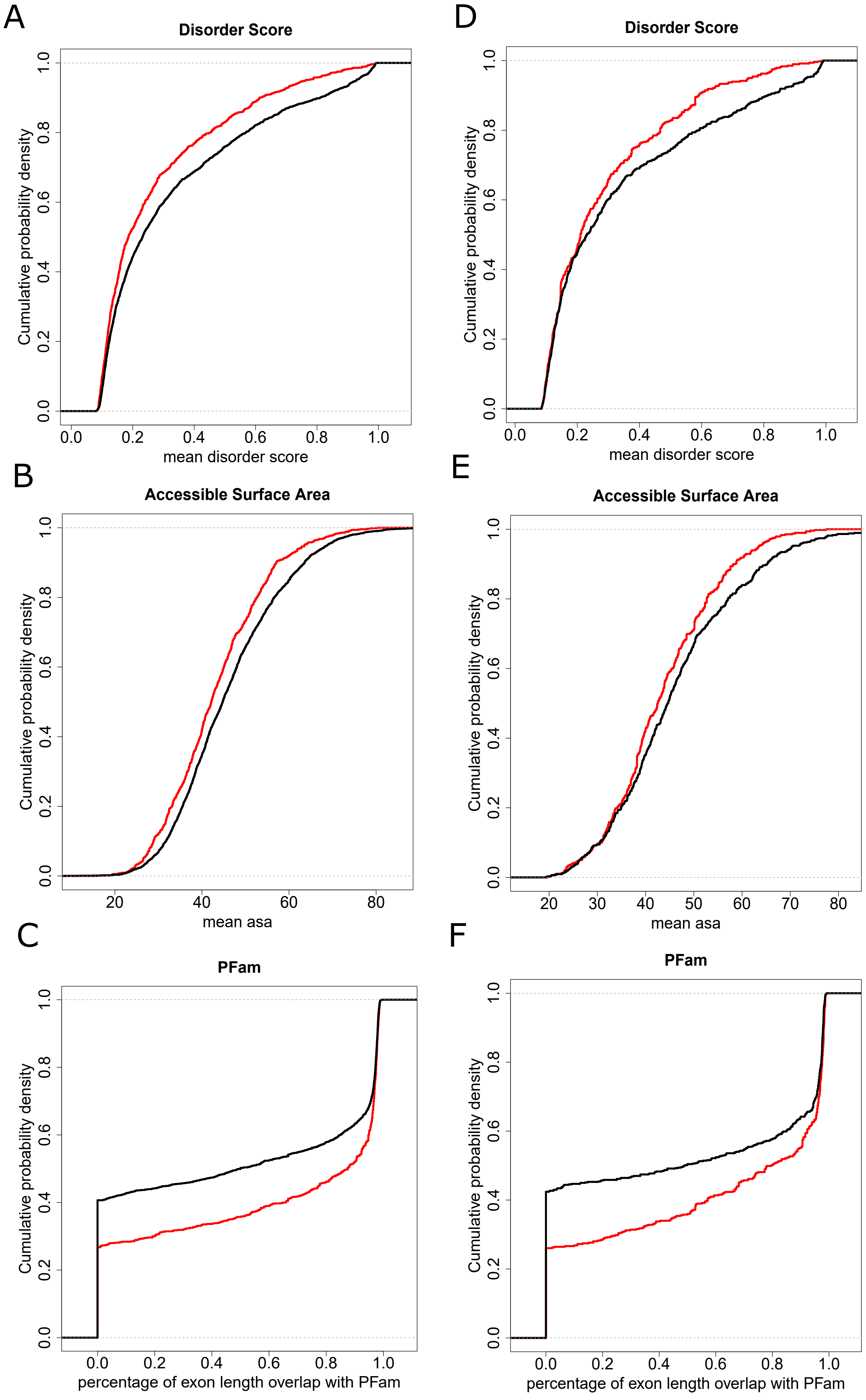
A
Disorder Score
Cumulative probability density
mean disorder score
0.0
0.2
0.4
0.6
0.8
1.0
D
Disorder Score
Cumulative probability density
mean disorder score
B
Accessible Surface Area
Cumulative probability density
mean asa
20
40
60
80
E
Accessible Surface Area
Cumulative probability density
mean asa
20
30
40
50
60
70
80
C
PFam
Cumulative probability density
percentage of exon length overlap with PFam
F
PFam
Cumulative probability density
percentage of exon length overlap with PFam

Fig S6.

A

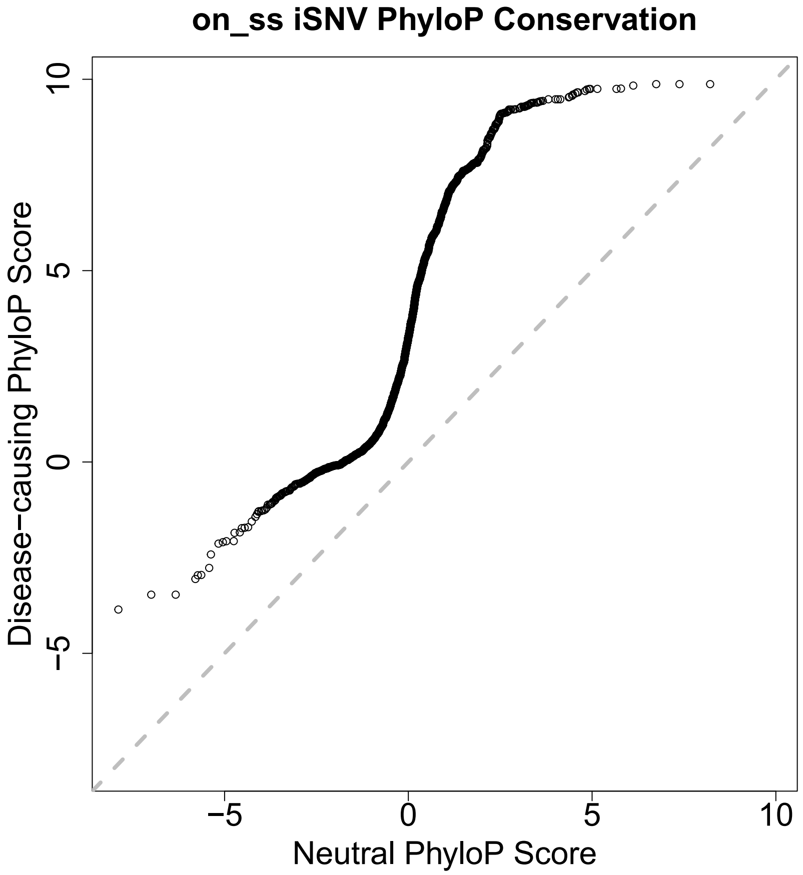
on_ss iSNV PhyloP Conservation
Disease−causing PhyloP Score
Neutral PhyloP Score
10
5
0
−5
−5
0
5
10

B

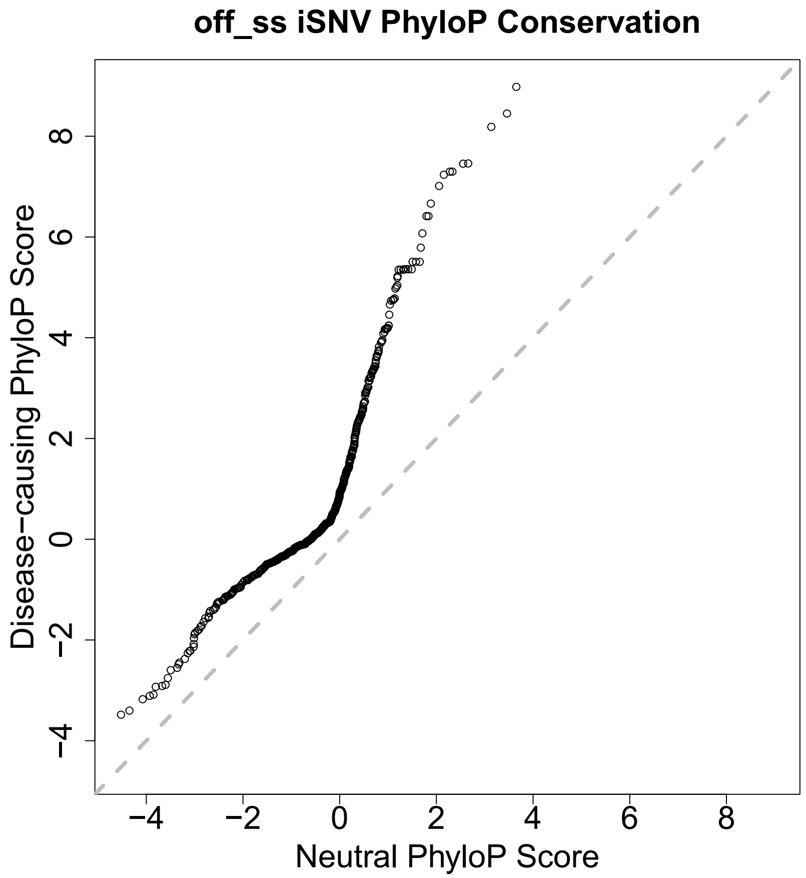
off_ss iSNV PhyloP Conservation
Disease−causing PhyloP Score
Neutral PhyloP Score
8
6
4
2
0
−2
−4
−4
−2
0
2
4
6
8

Fig S7.

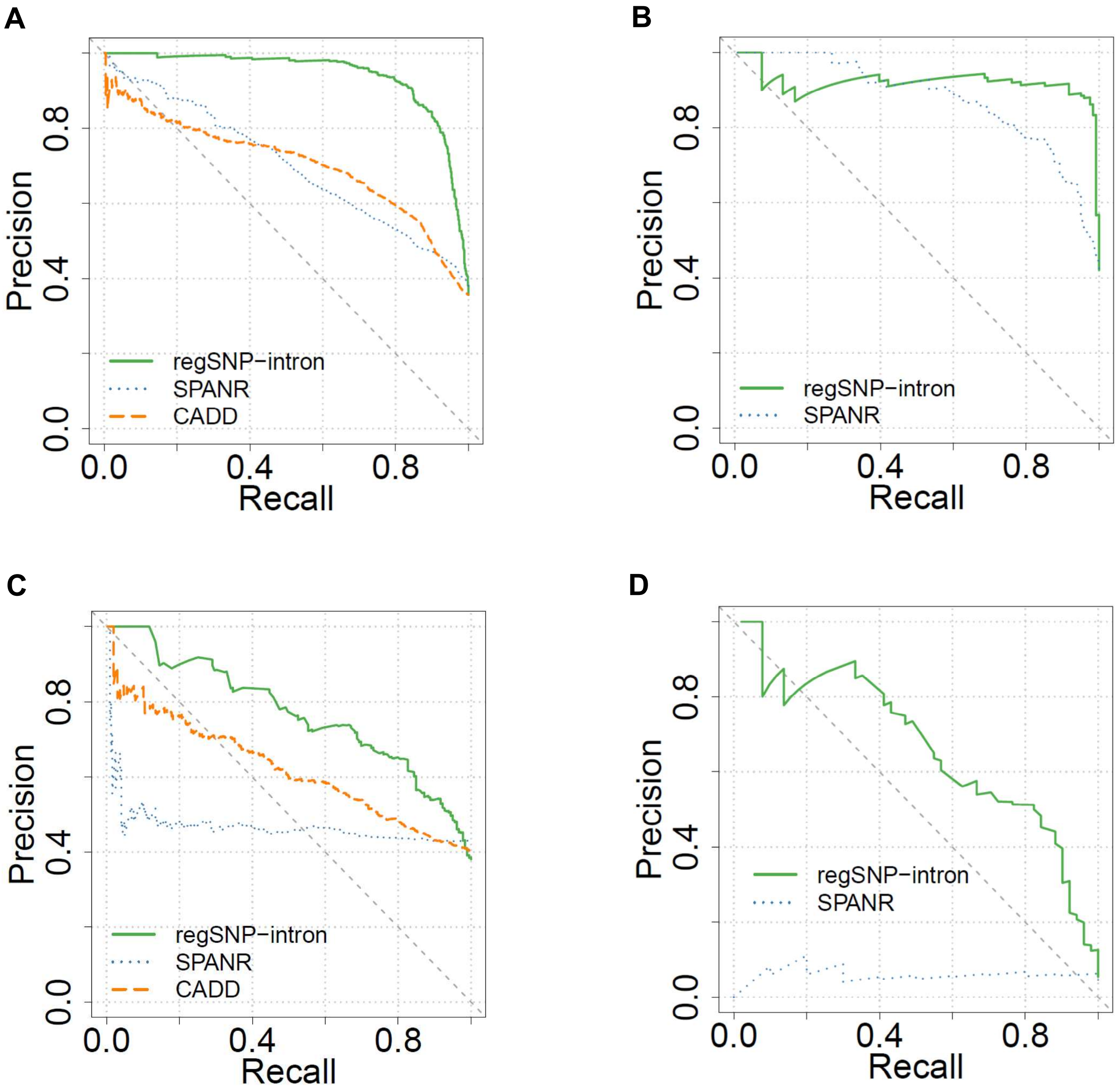

Fig S8.

A

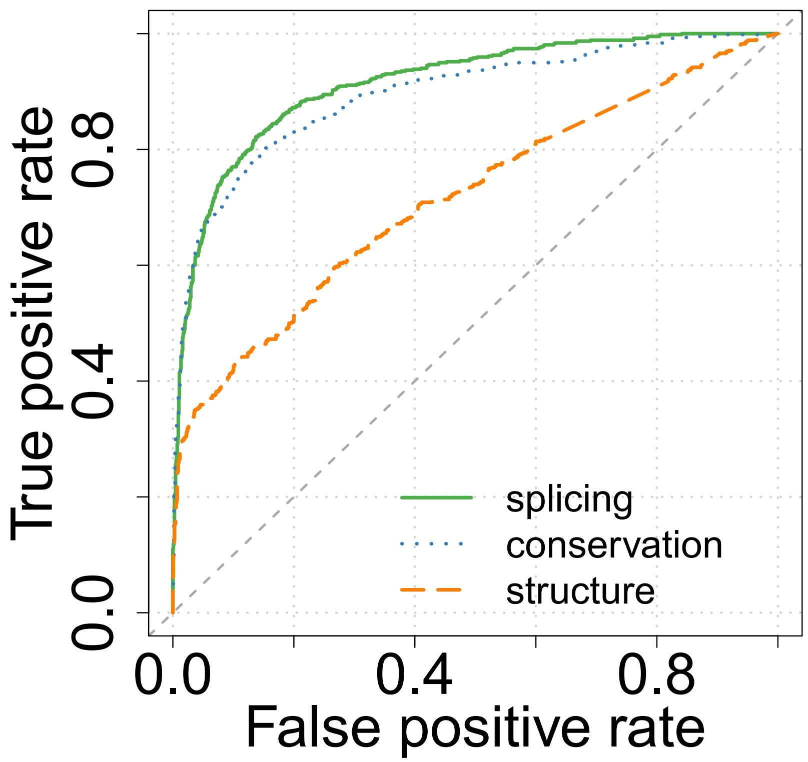

True positive rate
0.0
0.4
0.8
False positive rate
0.0
0.4
0.8
splicing
conservation
structure

B

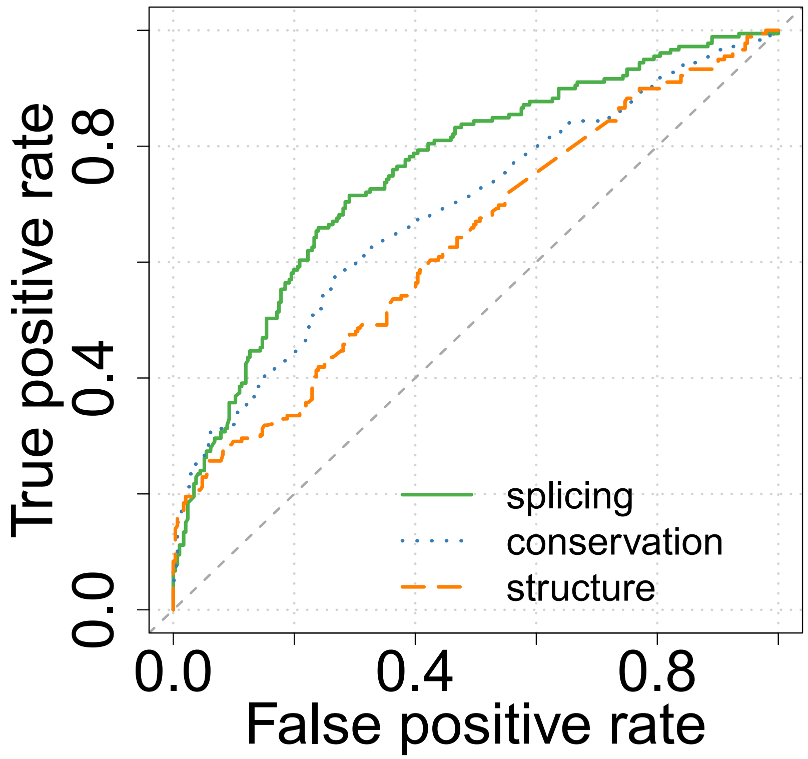

True positive rate
0.0
0.4
0.8
False positive rate
0.0
0.4
0.8
splicing
conservation
structure

Fig S9.

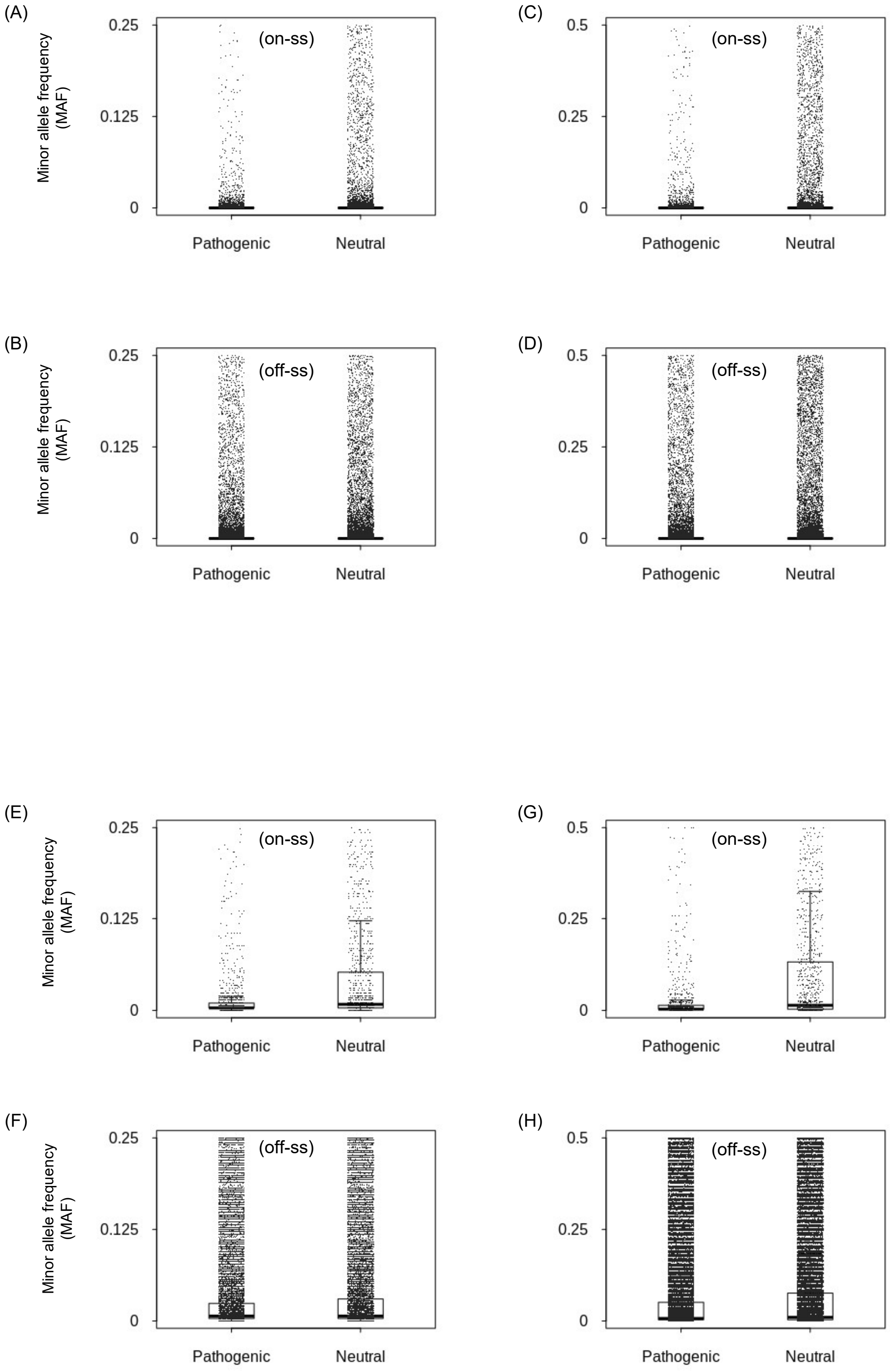

(A)
Minor allele frequency (MAF)
0.25
0.125
0
(on-ss)
Pathogenic
Neutral
(B)
Minor allele frequency (MAF)
0.25
0.125
0
(off-ss)
Pathogenic
Neutral
(C)
0.5
0.25
0
(on-ss)
Pathogenic
Neutral
(D)
0.5
0.25
0
(off-ss)
Pathogenic
Neutral
(E)
Minor allele frequency (MAF)
0.25
0.125
0
(on-ss)
Pathogenic
Neutral
(F)
Minor allele frequency (MAF)
0.25
0.125
0
(off-ss)
Pathogenic
Neutral
(G)
0.5
0.25
0
(on-ss)
Pathogenic
Neutral
(H)
0.5
0.25
0
(off-ss)
Pathogenic
Neutral

Fig S10.

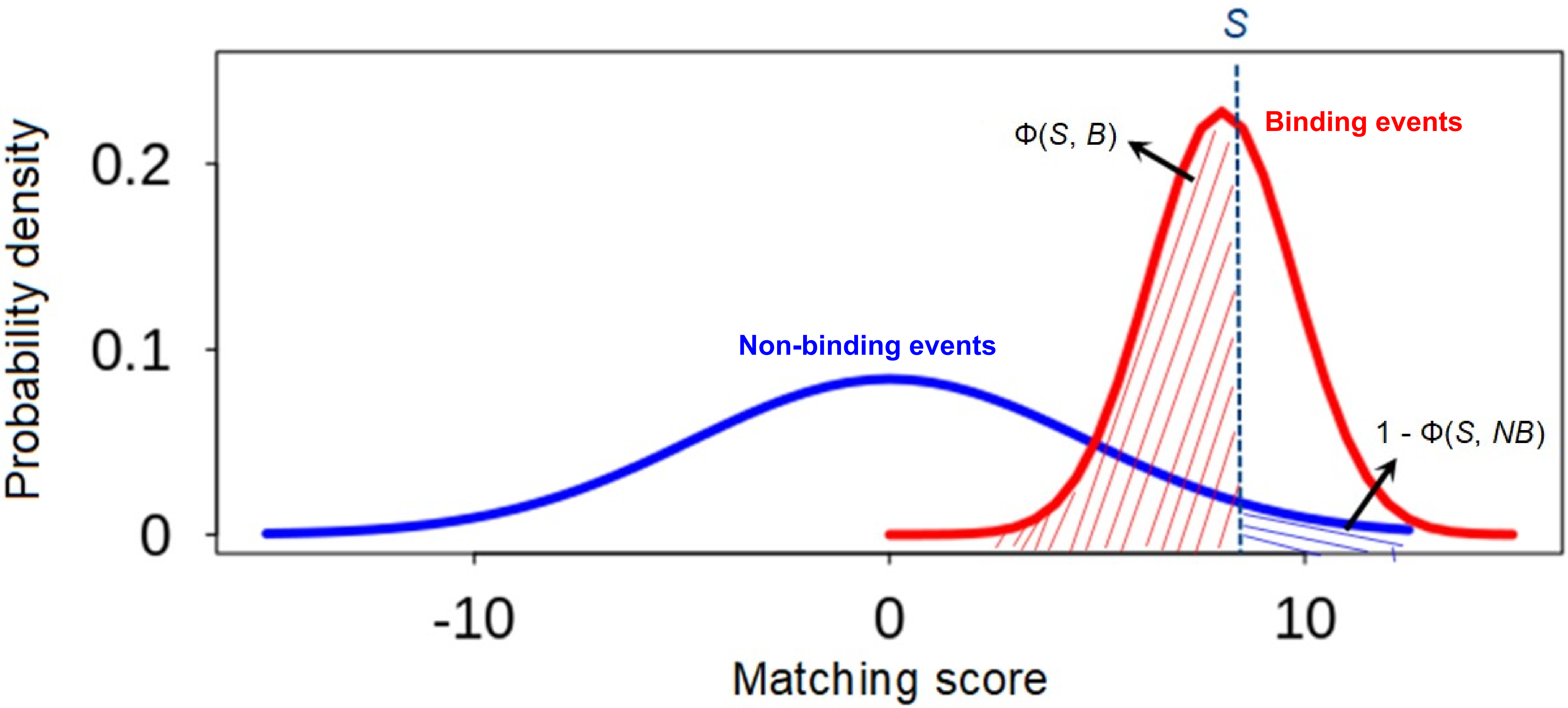
S
Φ(S, B)
Binding events
Non-binding events
1 - Φ(S, NB)
Probability density
0.2
0.1
0
-10
0
10
Matching score

SUPPLEMENTARY FIGURES

Figure S1. Detailed technical protocol.

Training data were collected from HGMD and the 1000 Genomes Project. iSNVs were first split into two classes, on-ss and off-ss respectively. 2/3 of the data were used for training, and the remaining 1/3 of data were used for validation. Features were extracted and two separate random forest classifiers were built and then used to predict the disease-causing probabilities for on-ss and off-ss iSNVs. An independent test set from the ClinVar database was also used for additional model validation. The prediction performance was separately evaluated for on-ss and off-ss iSNVs. *Genomic features*: features related to DNA/RNA, e.g. exon-intron junction score, RBP binding, PhyloP sequence conservation score; *structural features*: features related to protein domains or functions, e.g. ASA score, intrinsic disorder score, secondary structure score, PTM or Pfam score.

Figure S2. Data pre-processing.

Pathogenic iSNVs (HGMD, *top*) are closer to exons as compared to neutral iSNVs (1000 Genomes, *middle*). Thus, the distances from splice junction sites between on-ss and off-ss iSNVs were not balanced. Since the numbers of on-ss iSNVs were comparable in the pathogenic and neutral groups, the large number of off-ss neutral iSNVs needed to be down-sampled to avoid potential bias in machine learning. Down-sampled off-ss iSNV data are shown at the *bottom*.

Figure S3. Distribution of changes in splice-junction scores.

Pathogenic iSNVs (red) had significantly lower junction scores than neutral iSNVs (black) at both acceptor splice sites (A) and donor splice sites (B).

Figure S4. Average RBP binding score changes.

RBP binding score changes for on-ss (A) and off-ss (B) iSNVs. Each dot represents one RBP. The x-axis is the average RBP binding score change induced by pathogenic iSNVs (*binding score with alternative allele – binding score with reference allele*). The y-axis is the average RBP binding score change induced by neutral iSNVs.

Figure S5. Cumulative probability density of protein structural features.

Pathogenic on-ss (red, A) and off-ss (red, D) iSNVs had lower disorder scores than the neutral iSNVs (black in A and D), indicating that pathogenic iSNVs are more likely to be located near exons encoding structured peptide regions. In addition, pathogenic iSNVs (on-ss, red in B; off-ss, red in E) tend to be located near exons with smaller average ASA scores as compared to neutral iSNVs (black in B and E). Moreover, pathogenic iSNVs (on-ss and off-ss, red in C and F respectively) are also more likely to be located in the vicinity of exons encoding protein regions that overlap with Pfam domains.

Figure S6. Quantile-quantile plot of PhyloP conservation scores.

Pathogenic iSNVs are at loci that have significantly higher PhyloP scores compared to neutral iSNVs (on-ss, A; off-ss, B).

Figure S7. Model evaluation in terms of precision-recall curves.

(A - B): On-ss iSNVs. (A) Precision-recall curves (PRC) of regSNPs-intron (solid green), SPANR (dotted blue) and CADD (dashed orange) on the validation set. (B) PRC of regSNPs-intron (solid green) and SPANR (dotted blue) on the independent ClinVar test set. CADD was excluded since ClinVar was used in its model training. (C - D): Off-ss iSNVs, same as (A - B).

Figure S8. Performance of sub-models with features from each individual category.

Receiver operating characteristic curves (ROC) of models trained with features only from the splicing (green), protein structural (orange), and evolutionary conservation (blue) categories respectively. (A) on-ss iSNVs, (B) off-ss iSNVs.

Figure S9. Distribution of minor allele frequencies of ExAC and GTEx iSNVs.

Distribution of minor allele frequencies (MAF) of all iSNVs collected from ExAC (**A** - **D**) and GTEx (**E** - **H**), for on-ss and off-ss iSNVs in low (0 – 0.25) and medium (0 – 0.5) ranges of allele frequency. Pathogenic iSNVs were characterized by FPR < 0.1, and neutral ones were those with FPR ≥ 0.1. Since the number of neutral iSNVs was much greater than pathogenic iSNVs, we sampled an equal number of neutral iSNVs (with the lowest probabilities of pathogenicity) to compare with the pathogenic iSNVs in the plot to avoid visual bias. (**A**) on-ss iSNVs for allele frequency 0 – 0.25. (**B**) off-ss iSNVs for allele frequency 0 – 0.25. (**C**) on-ss iSNVs for allele frequency 0 – 0.5. (**D**) off-ss iSNVs for allele frequency 0 – 0.5. (**E** - **H**) same as (**A** - **D**).

Figure S10. Demonstration of PWM-derived matching score distribution.

The matching score for either RBP binding (red curve) or non-binding (blue curve) sites follow a Gaussian distribution. Shapes of the respective distributions (mean and variance) were determined by the actual data. For any matching score *S* derived from the PWM for a given RBP, the score for being a binding event equals to $\Phi(S, B)$, the red-shaded area under the red curve; whereas the score for being a non-binding event equals $1 - \Phi(S, NB)$, which is the blue-shaded area under the blue curve.
