# Supplementary material for: RegSNPs-intron: a computational framework for predicting pathogenic impact of intronic single nucleotide variants
Source: Genome Biol. 2019 Nov 28;20:254. doi: 10.1186/s13059-019-1847-4 (PMC6883696; doi:10.1186/s13059-019-1847-4)
Supplement: Supplementary file 4 — Additional file 4: Text S1. Sequence of the modified Exontrap plasmid in ASSET-seq. [file 13059_2019_1847_MOESM4_ESM.docx]

**Supplemental Text S1**

**Sequence of modified Exontrap plasmid with test insert (see also Figure 5A for graphic view)**

**KEY**

BARCODE

common region 1

exon homology (exon 1)

**BC (1 nt barcode: T-ref, A- alt)**

TEST EXON **SNP** TEST INTRON

intron homology

common region 2

exon homology 2

BARCODE

XXXXXXgcacctttgtggttctcacttggtggaagctctctacctggtgtgtggggagcgtggattcttctacacacccatgt **BC** TEST EXON SNP TEST INTRON tggagctcggtacctatttggggaccccatagagcactgcactgactgagggatggtaacaggatgtgtaggttttggaggcccata tgtccattcatgaccagtgacttgtctcacagccatgcaacccttgcctcctgtgctgacttagcaggggataaagtgagagaaagcctgggctaatcagggggtcgctcagctcctcctaactggattgtcctatgtgtctttgcttctgtgctgctgatgctctgccctgtgctgacatgacctccctggcagtggcacaactggagctgggtggaXXXXXX

**EXAMPLE**

AACGTCgcacctttgtggttctcacttggtggaagctctctacctggtgtgtggggagcgtggattcttctacacacccatgt**T**ACACCTAGGCCACTCGATCCCATGTCCTCGGCCTTCCCTCG**C**CCCTTTCTCACTCGTCCTCTCTACTTACCtggagctcggtacctatttggggaccccatagagcactgcactgactgagggatggtaacaggatgtgtaggttttggaggcccatatgtccattcatgaccagtgacttgtctcacagccatgcaacccttgcctcctgtgctgacttagcaggggataaagtgagagaaagcctgggctaatcagggggtcgctcagctcctcctaactggattgtcctatgtgtctttgcttctgtgctgctgatgctctgccctgtgctgacatgacctccctggcagtggcacaactggagctgggtggaCTCAAA

^*^Note: The *test exon* and *intron* is the unique part of the insert oligo. This is a fragment taken from the real gene hosting the SNP to be tested, including 11 bp of the closest exon on the 5’-side of the SNP and 60 bp of the intron containing the SNP.

ID barcodes for reads (FWD/REV in 5'-3' direction):

|  | **Input** | | **HeLa** | | **HEK293** | | **HepG2** | |
| --- | --- | --- | --- | --- | --- | --- | --- | --- |
| Replicate | Forward | Reverse | Forward | Reverse | Forward | Reverse | Forward | Reverse |
| **1** | AACGTC | AAACTC | ATCAAC | ATGCAC | GAAACC | GACAAT | TAGAAC | TATGCC |
| **2** | ACATGT | ACAACC | ATGTTG | CAATAC | GCACTA | GCGTTT | TCAAAG | TCCATA |
| **3** | ACCTTT | ACGGTT | CACAAG | CATCTA | GGTCTA | GTAATC | TCGATT | TCTACC |
| **4** | AGAAGG | AGACGT | CCAAAT | CGTTTC | GTAGAG | GTTAGT | TGCTAG | TGAACC |
| **5** | AGTGGA | AGTTAC | CTATGG | CTCCTT | TAACCC | TACAGA | TTCGAA | TTAACG |

**Plasmid Sequence:**

GGGGCGGAGAATGGGCGGAACTGGGCGGAGTTAGGGGCGGGATGGGCGGAGTTAGGGGCGGGACTATGGTTGCTGACTAATTGAGATGCATGCAAGGAGATGGCGCCCAACAGTCCCCCGGCCACGGGGCCTGCCACCATACCCACGCCGAAACAAGCGCTCATGAGCCCGAAGTGGCGAGCCCGATCTTCCCCATCGGTGATGTCGGCGATATAGGCGCCAGCAACCGCACCTGTGGCGCCGGTGATGCCGGCCACGATGCGTCCGGCGTAGAGGATCTCAGGATATAGTAGTTTCGCTTTTGCATAGGGAGGGGGAAATGTAGTCTTATGCAATACTCTTGTAGTCTTGCAACATGCTTATGTAACGATGAGTTAGCAACATGCCTTATAAGGAGAGAAAAAGCACCGTGCATGCCGATTGGTGGGAGTAAGGTGGTATGATCGTGGTATGATCGTGCCTTGTTAGGAAGGCAACAGACGGGTCTAACACGGATTGGACGAACCACTGAATTCCGCATTGCAGAGATATTGTATTTAAGTGCCTAGCTCGATACAATAAACGCCATTTGACCATTCACCACATTGGTGTGCACCTCAAGCTTCCTGCATGCTGCTGCTGCTGCTGCTGCTGGGCCTGAGGCTACAGCTCTCCCTGGGCATCATCCCAGTTGAGGAGGAGAACCCGGACTTCTGGAACCGCGAGGCAGCCGAGGCCCTGGGTGCCGCCAAGAAGCTGCAGCCTGCACAGACAGCCGCCAAGAACCTCATCATCTTCCTGGGCGATGGGATGGGGGTGTCTACGGTGACAGCTGCCAGGATCGATCCGCTTCCTGCCCCTGCTGGCCCTGCTCATCCTCTGGGAGCCCCGCCCTGCCCAGGCTTTTGTCAAACAGCACCTTTGTGGTTCTCACTTGGTGGAAGCTCTCTACCTGGTGTGTGGGGAGCGTGGATTCTTCTACACACCCATGTACCGGTATTCAGCCGCTAGCTGGAGCTCGGTACCTATTTGGGGACCCCATAGAGCACTGCACTGACTGAGGGATGGTAACAGGATGTGTAGGTTTTGGAGGCCCATATGTCCATTCATGACCAGTGACTTGTCTCACAGCCATGCAACCCTTGCCTCCTGTGCTGACTTAGCAGGGGATAAAGTGAGAGAAAGCCTGGGCTAATCAGGGGGTCGCTCAGCTCCTCCTAACTGGATTGTCCTATGTGTCTTTGCTTCTGTGCTGCTGATGCTCTGCCCTGTGCTGACATGACCTCCCTGGCAGTGGCACAACTGGAGCTGGGTGGAGGCCCGTGACCTTCAGACCTTGGCACTGGAGGTGGCCCGGCAGAAGCGCGGCATCGTGGATCAGTGCTGCACCAGCATCTGCTCTCTCTACCAACTGGAGAACTACTGCAACTAGGCCCACCACTACCCTGTCCACCCCTCTGCAATGAATAAAACCTTTGAAAGAGCACTACAAGTTGTGTGTACATGCGTGCATGTGCATATGTGGTGCGGGGGGAACATGAGTGGGGCTGGCTGGAGTGGCGATGATAAGCTGTCAAACATGAGAATTCTTGAAGACGAAAGGGCCTCGTGATACGCCTATTTTTATAGGTTAATGTCATGATAATAATGGTTTCTTAGACGTCAGGTGGCACTTTTCGGGGAAATGTGCGCGGAACCCCTATTTGTTTATTTTTCTAAATACATTCAAATATGTATCCGCTCATGAGACAATAACCCTGATAAATGCTTCAATAATATTGAAAAAGGAAGAGTATGAGTATTCAACATTTCCGTGTCGCCCTTATTCCCTTTTTTGCGGCATTTTGCCTTCCTGTTTTTGCTCACCCAGAAACGCTGGTGAAAGTAAAAGATGCTGAAGATCAGTTGGGTGCACGAGTGGGTTACATCGAACTGGATCTCAACAGCGGTAAGATCCTTGAGAGTTTTCGCCCCGAAGAACGTTTTCCAATGATGAGCACTTTTAAAGTTCTGCTATGTGGCGCGGTATTATCCCGTGTTGACGCCGGGCAAGAGCAACTCGGTCGCCGCATACACTATTCTCAGAATGACTTGGTTGAGTACTCACCAGTCACAGAAAAGCATCTTACGGATGGCATGACAGTAAGAGAATTATGCAGTGCTGCCATAACCATGAGTGATAACACTGCGGCCAACTTACTTCTGACAACGATCGGAGGACCGAAGGAGCTAACCGCTTTTTTGCACAACATGGGGGATCATGTAACTCGCCTTGATCGTTGGGAACCGGAGCTGAATGAAGCCATACCAAACGACGAGCGTGACACCACGATGCCTGCAGCAATGGCAACAACGTTGCGCAAACTATTAACTGGCGAACTACTTACTCTAGCTTCCCGGCAACAATTAATAGACTGGATGGAGGCGGATAAAGTTGCAGGACCACTTCTGCGCTCGGCCCTTCCGGCTGGCTGGTTTATTGCTGATAAATCTGGAGCCGGTGAGCGTGGGTCTCGCGGTATCATTGCAGCACTGGGGCCAGATGGTAAGCCCTCCCGTATCGTAGTTATCTACACGACGGGGAGTCAGGCAACTATGGATGAACGAAATAGACAGATCGCTGAGATAGGTGCCTCACTGATTAAGCATTGGTAACTGTCAGACCAAGTTTACTCATATATACTTTAGATTGATTTAAAACTTCATTTTTAATTTAAAAGGATCTAGGCTGCTGCTTGCAAACAAAAAAACCACCGCTACCAGCGGTGGTTTGTTTGCCGGATCAAGAGCTACCAACTCTTTTTCCGAAGGTAACTGGCTTCAGCAGAGCGCAGATACCAAATACTGTCCTTCTAGTGTAGCCGTAGTTAGGCCACCACTTCAAGAACTCTGTAGCACCGCCTACATACCTCGCTCTGCTAATCCTGTTACCAGTGGCTGCTGCCAGTGGCGATAAGTCGTGTCTTACCGGGTTGGACTCAAGACGATAGTTACCGGATAAGGCGCAGCGGTCGGGCTGAACGGGGGGTTCGTGCACACAGCCCAGCTTGGAGCGAACGACCTACACCGAACTGAGATACCTACAGCGTGAGCTATGAGAAAGCGCCACGCTTCCCGAAGGGAGAAAGGCGGACAGGTATCCGGTAAGCGGCAGGGTCGGAACAGGAGAGCGCACGAGGGAGCTTCCAGGGGGAAACGCCTGGTATCTTTATAGTCCTGTCGGGTTTCGCCACCTCTGACTTGAGCGTCGATTTTTGTGATGCTCGTCAGGGGGGCGGAGCCTATGGAAAAACGCCAGCAACGGAGATGCGCCGCGTGCGGCTGCTGGAGATGGCGGACGCGATGGATATGTTCTGCCAAGGGTTGGTTTGCGCATTCACAGTTCTCCGCAAGAATTGATTGGCTCCAATTCTTGGAGTGGTGAATCCGTTAGCGAGGTGCCGCCGGCTTCCATTCAGGTCGAGGTGGCCCGGCTCCATGCACCGCGACGCAACGCGGGGAGGCAGACAAGGTATAGGGCGGCGCCTACAATCCATGCCAACCCGTTCCATGTGCTCGCCGAGGCGGCATAAATCGCCGTGACGATCAGCGGTCCAATGATCGAAGTTAGGCTGGTAAGAGCCGCGAGCGATCCTTGAAGCTGTCCCTGATGGTCGTCATCTACCTGCCTGGACAGCATGGCCTGCAACGCGGGCATCCCGATGCCGCCGGAAGCGAGAAGAATCATAATGGGGAAGGCCATCCAGCCTCGCGTCGGGGAGCTTTTTGCAAAAGCCTAGGCCTCCAAAAAAGCCTCCTCACTACTTCTGGAATAGCTCAGAGGCCGAGGCGGCCTCGGCCTCTGCATAAATAAAAAAAATTAGTCAGCCAT
