# Supplementary material for: RegSNPs-intron: a computational framework for predicting pathogenic impact of intronic single nucleotide variants
Source: Genome Biol. 2019 Nov 28;20:254. doi: 10.1186/s13059-019-1847-4 (PMC6883696; doi:10.1186/s13059-019-1847-4)
Supplement: Supplementary file 5 — Additional file 5. Review history. [file 13059_2019_1847_MOESM5_ESM.docx]

Review History

**First round of review**

**Reviewer 1**

**Were you able to assess all statistics in the manuscript, including the appropriateness of statistical tests used?**

yes

**Comments to author:**

In this paper, Lin et al. developed the regSNPs-intron algorithm based on a random forest classifier that integrates RNA splicing, protein structure and evolutionary conservation features. The goal is to tell whether an intro SNV (named iSNV) is pathogenic. The author also conducted experiments to verify their findings. This is an interesting piece of work on an important problem. However, the description of their method is quite confusing in multiple places. 

1.     The title is a bit misleading. From my understanding, regSNPs-intron is a random forest-based classifier to decide whether an iSNV is pathogenic or neutral. So it is for pathogenic prediction. It is not elaborated how to prioritize SNVs using this tool. I suggest revising the title to reflect the true content of the paper.
2.     In Abstract, line 18-20, "regSNPs-intron showed high accuracy in computing disease-causing probabilities of iSNVs." I found this statement to be rather confusing. I don't think there is such thing called "disease-causing probability". It is not defined and I doubted it can be defined at all. Hence it is meaningless to talk about "accuracy". 
3.     In Abstract, line 23-25, "we validated regSNPs-intron predictions by measuring the impact of iSNVs on splicing outcome." Since I believe splicing outcome may not directly imply disease pathogenicity, I am not sure "validated" is the right word to use here.
4.     The first paragraph in the background section is misleading. My understanding is that coding variants only accounts for about 1 or 2 percent of all variants identified by GWAS. Among all the non-coding variants, roughly half of them are located in introns and half of them located intergenic. To be fair, the authors need to acknowledge there are substantial number of variants located outside the boundary of any gene. So iSNVs are not the only variants that are not located in exons.   
5.     There are a rich set of literature on non-coding variant annotation and function prediction. It is highly recommended that a more comprehensive literature review to be conducted on the existing methods. See a recent review on this topic (Rojano et al. Brief Bioinform 2018).
6.     How many pathogenic iSNVs and neutral iSNVs from HGMD and ClinVAR respectively are used in the test? I got a feeling very imbalanced test sets are used. If this is the case, AUPR performance is more meaningful than AUC. Alternatively  the authors can choose to down sample the neutral iSNV set.
7.     Figure 2. Why there is a hard threshold (at 10^-10) for FDR? Also, I am not sure about the feature scores. Is there a score for every feature? And all of these features are ranged 0 to 1? I am not able to find detailed description of these plotted feature scores in the Method section. Is it true that the direction of the scores is not meaningful? If so, it is recommended to plot the absolute value of score differences.
8.     Figure 3. Part (B). there is no figure legend. And the color scheme is the same as (A) and (C), but the meanings are completely different. This is very confusing. 
9.     Figure 3. Part (D). When allele frequency change from 0.1 to 0.9, the allele changes from mutant to wild type, and it is obvious that the significant difference in pathogenicity probability. I think one should focus on the minor allele, and plot probability against minor allele frequency instead.
10.     Figure 4. I assumed these iSNVs are on-ss. The range for the probability in this plot is really tight. However in Figure 3(D), the range for on-SS iSNVs are much bigger, why the difference?
11.     Page 19, formula for likelihood ratio M, it is not explained why the probability ratio P(S_A|B)/P(S_A|NB) is equal to the integral. 
12.     Page 20, formula for posterior probability change P, this formula does not make any sense! d(B), integrate over B? B is an event, not a continuous variable takes value from 0 to 1. 
13.     Page 24. The regression equation also does not make any sense. Y is discrete. A generalized linear model should be used here with a link function based on the negative binomial distribution.
14.     Page 21. "GTEx and ExAC database iSNVs." How many on-ss iSNVs and off-ss iSNVs are from ExAC? 
15.     Figure 2. Color dots in the figure legend are mixed with dots in the figure, suggest a box to plot around the figure legend to avoid confusion.
16.     Why these three particular cell lines were selected?

**Reviewer 2**

**Were you able to assess all statistics in the manuscript, including the appropriateness of statistical tests used?**

yes

**Comments to author:**

In this manuscript, the authors applied random forest (RF) classifier, called regSNP-intron, to predict (and prioritize) the pathogenic intronic SNV (iSNV) from the background of neutral iSNV. There are three categories of features are used for the prediction: splicing related, protein structure related, and evolution related features. Each type of these features is shown to positively or negatively correlated to the pathogenic state of the iSNV, and hence used to predict the pathogenic state and achieved superior performance comparing to the two alternative methods SPANR and CADD. 

Overall, it is an interesting question to address and the manuscript is very well written. However, I have two major concerns on the procedure of the machine learning settings and benchmarking with other methods.

1) The number of negative (neutral) samples for the off-ss iSNV is not clear. Is it the total 2,104,613 off-ss neutral iSNVs or the subsampled 852 samples? If in the first case, the positive (pathogenic) and negative (neutral) samples are largely imbalanced. Even in the latter case, there still is a minor imbalance and can bias the evaluation, which is the same for the on-ss iSNV. In either case, it would be important to see the performance with a balanced training set, as RF is sensitive to the label balance. One possible way to down sample the negative set to the same size of the positive samples and train the RF model on it. Multiple repeats can be used by re-sampling the negative samples for full use of the data.

2) It is not clear how the alternative methods SPANR and CADD are used, and which makes it very difficult to understand what contributes to the improvement of regSNP-intron comparing to the alternatives. From what the text implies, the authors used SPANR and CADD as a pre-trained system and directly tested the validation and test sets. If this is true, I doubt that the improved performance may be largely contributed by the better training set (e.g., the sample bias as above), instead of the better features nor the RF model. Actually, with only splicing features or the three evolutionary features, regSNP-intron is already largely better than SPANR and CADD. This surprised me, as I believe at least SPANR uses the evolutionary features and part of the splicing features. Also, it is not likely that RF performs so much better than neural network used in SPANR on this task.


A few minor comments:
- Fig 1: please add the number of features in each category, e.g., how many for splicing-junction score, and BRP binding score
- Table S1: need more description on each feature
- Fig 3D & 3H: why introduce 20 bins instead use each dot for an iSNV. Here, each dot denotes a set of iSNV in a bin, but the number of iSNV in each bin can be different. One supplementary figure with the original instead of binned iSNV would help.
- Fig 4: not sure what each dot denotes? Is it an exon for each dot? If so, why so few, as 75,119 exons are mentioned in line 11 page 12?
- In the results section for "drug-induced cytotoxicity", only the pathogenic iSNV has been reported. How about the neutral iSNVs with the similar distance to the splice junction?

**Authors Response**

**Point-by-point responses to the reviewers’ comments:**

Reviewer #1: In this paper, Lin et al. developed the regSNPs-intron algorithm based on a random forest classifier that integrates RNA splicing, protein structure and evolutionary conservation features. The goal is to tell whether an intro SNV (named iSNV) is pathogenic. The author also conducted experiments to verify their findings. This is an interesting piece of work on an important problem. However, the description of their method is quite confusing in multiple places. 

1. The title is a bit misleading. From my understanding, regSNPs-intron is a random forest-based classifier to decide whether an iSNV is pathogenic or neutral. So it is for pathogenic prediction. It is not elaborated how to prioritize SNVs using this tool. I suggest revising the title to reflect the true content of the paper. 

*Response: We thank the reviewer for the suggestion. We have modified our title to be “RegSNPs-Intron: a computational framework for predicting pathogenic impact of intronic single nucleotide variants”.* 

2. In Abstract, line 18-20, "regSNPs-intron showed high accuracy in computing disease-causing probabilities of iSNVs." I found this statement to be rather confusing. I don't think there is such thing called "disease-causing probability". It is not defined and I doubted it can be defined at all. Hence it is meaningless to talk about "accuracy". 

*Response: We have avoided using “disease-causing probability” according to reviewer’s suggestion. The original statement is now changed to: “regSNPs-intron showed excellent performance in evaluating the pathologic impact of iSNVs". See the Abstract in the revised manuscript for the update.* 

3. In Abstract, line 23-25, "we validated regSNPs-intron predictions by measuring the impact of iSNVs on splicing outcome." Since I believe splicing outcome may not directly imply disease pathogenicity, I am not sure "validated" is the right word to use here. 

*Response: We appreciate the reviewer’s comments. In the revised manuscript, we have modified the text accordingly. The original statement is now changed to: “Using a high-throughput functional reporter assay called ASSET-seq (ASsay for Splicing using ExonTrap and sequencing), we evaluated the impact of regSNPs-intron predictions on splicing outcome”. Please check the revised manuscript for the updated Abstract. In addition, we also revised a paragraph in the Discussion section (Page 18, Paragraph 1) addressing this issue.* 

4. The first paragraph in the background section is misleading. My understanding is that coding variants only accounts for about 1 or 2 percent of all variants identified by GWAS. Among all the non-coding variants, roughly half of them are located in introns and half of them located intergenic. To be fair, the authors need to acknowledge there are substantial number of variants located outside the boundary of any gene. So iSNVs are not the only variants that are not located in exons. 

*Response: We have followed the reviewer’s suggestion and updated the related text in the Background section in the revised manuscript (see Page 3, Paragraph 1). As suggested by reviewer, we have discussed the functions of both intronic and intergenic SNVs as non-coding variants.* 

5. There are a rich set of literature on non-coding variant annotation and function prediction. It is highly recommended that a more comprehensive literature review to be conducted on the existing methods. See a recent review on this topic (Rojano et al. Brief Bioinform 2018). 

*Response: We appreciate the reviewer’s advice, and hence provide a more comprehensive review on the existing methods/tools of both non-coding variant annotation and pathogenicity prediction. Please refer to the updated text in Page 3, Paragraph 1 and 2 in the Background section.* 

6. How many pathogenic iSNVs and neutral iSNVs from HGMD and ClinVAR respectively are used in the test? I got a feeling very imbalanced test sets are used. If this is the case, AUPR performance is more meaningful than AUC. Alternatively the authors can choose to down sample the neutral iSNV set. 

*Response: The total numbers of pathogenic and neutral iSNVs from HGMD (and 1000 Genomes) and ClinVar used in the model test are provided as the following below, and in the Result section (Page 6, Paragraph 2 and 3). While selecting the neutral iSNV sets, we have taken the same down-sampling approach as suggested by the reviewer to ensure the positive and negative samples in the training and testing sets are balanced. The detailed down-sampling method is described in Page 6, Paragraph 2. 

HGMD (and 1000 Genomes) 
pathogenic on-ss iSNVs: 622 
neutral on-ss iSNVs: 1129 
pathogenic off-ss iSNVs 191 
neutral off-ss iSNVs 284 

ClinVar 
pathogenic on-ss iSNVs 121 
neutral on-ss iSNVs 167 
pathogenic off-ss iSNVs 51 
neutral off-ss iSNVs 883 

In addition, as suggested by the reviewer, we have provided the precision-recall curves (PRC) to visualize the performances on both the test dataset (i.e. validation) and ClinVar dataset (i.e. independent test) respectively. The precision recall curves and the AUPRs are now included in Supplementary Figure S7.* 

7. Figure 2. Why there is a hard threshold (at 10^-10) for FDR? Also, I am not sure about the feature scores. Is there a score for every feature? And all of these features are ranged 0 to 1? I am not able to find detailed description of these plotted feature scores in the Method section. Is it true that the direction of the scores is not meaningful? If so, it is recommended to plot the absolute value of score differences. 

*Response: The FDRs for a substantial number of features have extremely small p values (original p-values can be found in Table S1). Hence, here the plot range of FDR was capped at 10-10 for ease of display. The score for any feature in each respective category of RNA splicing, protein structure, or evolutionary conservation (see also Figure 1) was calculated based on the descriptions in the Method section (see text from Pages 20 Paragraph 1 to Page 23 Paragraph 2). In the plot, every dot represents a feature. The x-axis is the feature’s delta median, calculated as the “median score of pathogenic iSNVs” minus the “median score of neutral iSNVs” of the particular feature; the y-axis is the significance value of the score difference based on Wilcoxon rank-sum test. Due to the large dynamic ranges of the feature values, the delta median values were rescaled to range [-1, 1]: x'=2(x-min(x))/(max(x)-min(x))-1. We have also clarified these in the revised manuscript (Figure Legends “Figure 2”, Page 33). Directions (i.e. arithmetic signs) for the values here might have different implications depending on the specific features. However, as pointed out by this reviewer, they might not be meaningful, particularly since our aim was to select the features that were significantly different (in terms of scores) between the two classes of iSNVs, rather than to interpret the exact implication for each feature. Thus, we have updated the plot using the absolute values of the score changes as recommended by reviewer (see the updated Figure 2 of the revised manuscript).* 

8. Figure 3. Part (B). there is no figure legend. And the color scheme is the same as (A) and (C), but the meanings are completely different. This is very confusing. 

*Response: We have changed the color setting in Figure 3B and 3F (i.e. new Figure index 3B and 3E in the update version) to black and white for a better clarity, so as to avoid confusion. Also, we have updated the legends for these figures (Page 33, “Figure 3”).* 

9. Figure 3. Part (D). When allele frequency change from 0.1 to 0.9, the allele changes from mutant to wild type, and it is obvious that the significant difference in pathogenicity probability. I think one should focus on the minor allele, and plot probability against minor allele frequency instead. 

*Response: We have considered the reviewer’s comment, and we agree that focusing on the minor allele when plotting the probability versus allele frequency will be a better way to indicate the relationship between pathogenic probability and allele frequency (AF). Thus, we have re-analyzed the correlation in two AF scales: 0.0 to 0.25 (low AF) and 0.0 to 0.5 (medium AF). As expected, negative correlations between the pathogenic probability and AF were observed in both scales for on-ss and off-ss iSNVs. Please see the new figures (Figure 4A - 4D) as well as the updated text in Page 12, Paragraph 2. We have also modified the figure legend text accordingly (Page 34, “Figure 4”).* 

10. Figure 4. I assumed these iSNVs are on-ss. The range for the probability in this plot is really tight. However in Figure 3(D), the range for on-SS iSNVs are much bigger, why the difference? 

*Response: In Figure 4, the iSNVs include both on-ss and off- ones. The purpose of the figure is to see whether there’s a trend that important exons co-locate fewer iSNVs in nearby, an indication of essentiality. iSNVs are those within ±300 bp of exon-intron boundaries, which are supposed to include both on-ss and off-ss iSNVs (see Page 13, Paragraph 2). The disease-related importance of an exon is scored as the probability calculated using one randomly-selected iSNV nearby (within ±300 bp from the boundary, could be either on-ss or off-ss). On the other hand, the range for the probability values of off-ss iSNVs is much smaller than that for on-ss iSNVs (see the new Figure 4A and 4C and also Figure 4B and 4D). The reason why the range was so tight in Figure 4 (i.e. Figure 4E in the new index) was because the number of off-ss iSNVs (from ExAC) was much larger than on-ss iSNVs (see also the response to review question #14 below). Thus, the results from off-ss iSNVs dominated the overall value. Nonetheless, the negative correlation between the predicted pathogenic score and number of iSNVs near the exon to be examined stands.* 

11. Page 19, formula for likelihood ratio M, it is not explained why the probability ratio P(S_A|B)/P(S_A|NB) is equal to the integral. 

*Response: We apologize for the confusion. We realized that the formula for calculating the log-likelihood ratio M was incorrect and that two other integral terms were missing in the formula. Specifically, the probability terms P(SA or R|B) and P(SA or R|NB) were assumed to follow a Gaussian distribution with respect to the particular mean (Ms) and variance (Vs) calculated in the way defined in the Methods (Page 21, Paragraph 1). Thus, each probability term is equal to one integral with respect to the density function. And as pointed out by reviewer, P(SA|B)/P(SA|NB) should not be equal to that one integral, but should be the ratio (or quotient) between two Gaussian-like integrals. We have accordingly updated that formula as well as the related text in Page 21, Paragraph 2 (Methods) in the revised paper. We appreciate the reviewer for pointing this out.* 

12. Page 20, formula for posterior probability change P, this formula does not make any sense! d(B), integrate over B? B is an event, not a continuous variable takes value from 0 to 1. 

*Response: We have updated the formula here to avoid confusion and misunderstanding. We have also put in additional notations on the formula terms to facilitate understanding. See Page 22, Paragraph 2 for the updated description.* 

13. Page 24. The regression equation also does not make any sense. Y is discrete. A generalized linear model should be used here with a link function based on the negative binomial distribution. 

*Response: We have double checked the regression equation. The response variable Y is the sequencing read count, which follows a negative binomial distribution (see Page 26, Paragraph 3). Complying with the GLM model suggested by the reviewer, we used an extended version, Generalized Linear Mixed Effect Model (GLMM) to account for the random effect associated with experimental variables (sample replicates). In the revised manuscript, we have clarified this at Page 26, Paragraph 3. See also in the same paragraph for notations on the equation terms.* 

14. Page 21. "GTEx and ExAC database iSNVs." How many on-ss iSNVs and off-ss iSNVs are from ExAC? 

*Response: There are 368,489 on-ss iSNVs and 2,314,839 off-ss iSNVs from ExAC. These numbers have been added in the Methods section (see Page 23, Paragraph 5).* 

15. Figure 2. Color dots in the figure legend are mixed with dots in the figure, suggest a box to plot around the figure legend to avoid confusion. 

*Response: We have adopted the reviewer’s suggestion and added a box around the legend. Please see the newly updated Figure 2.* 

16. Why these three particular cell lines were selected? 

*Response: We chose three cell lines that originated from different tissues. This served to potentially illustrate the variability in splicing activity between cell lines. The liver and kidney cell lines reflected our interest in liver and kidney function. In addition, all three cell lines are commonly used in many research laboratories, which facilitates both replication by other labs and potentially linking the splicing results with previously conducted molecular analyses.* 


Reviewer #2: In this manuscript, the authors applied random forest (RF) classifier, called regSNP-intron, to predict (and prioritize) the pathogenic intronic SNV (iSNV) from the background of neutral iSNV. There are three categories of features are used for the prediction: splicing related, protein structure related, and evolution related features. Each type of these features is shown to positively or negatively correlated to the pathogenic state of the iSNV, and hence used to predict the pathogenic state and achieved superior performance comparing to the two alternative methods SPANR and CADD. 

Overall, it is an interesting question to address and the manuscript is very well written. However, I have two major concerns on the procedure of the machine learning settings and benchmarking with other methods. 

1) The number of negative (neutral) samples for the off-ss iSNV is not clear. Is it the total 2,104,613 off-ss neutral iSNVs or the subsampled 852 samples? If in the first case, the positive (pathogenic) and negative (neutral) samples are largely imbalanced. Even in the latter case, there still is a minor imbalance and can bias the evaluation, which is the same for the on-ss iSNV. In either case, it would be important to see the performance with a balanced training set, as RF is sensitive to the label balance. One possible way to down sample the negative set to the same size of the positive samples and train the RF model on it. Multiple repeats can be used by re-sampling the negative samples for full use of the data. 

*Response: The number of negative (neutral) samples for off-ss iSNVs was 852. As described at Page 6, Paragraph 2 in the Results subsection Datasets, we performed down-sampling to 1) balance the positive and negative samples, and 2) avoid the bias introduced by varying distances of iSNVs to junction sites (see also Supplemental Figure S2). The performed down-sampling specifically ensured that the neutral off-ss iSNVs had a similar distribution as the pathogenic off-ss iSNVs. Hence, the numbers (pathogenic vs. neutral = 573 vs. 852) as well as the numerical properties for both sample sets were more balanced (see also Question #6 in Reviewer #1 above).* 

2) It is not clear how the alternative methods SPANR and CADD are used, and which makes it very difficult to understand what contributes to the improvement of regSNP-intron comparing to the alternatives. From what the text implies, the authors used SPANR and CADD as a pre-trained system and directly tested the validation and test sets. If this is true, I doubt that the improved performance may be largely contributed by the better training set (e.g., the sample bias as above), instead of the better features nor the RF model. Actually, with only splicing features or the three evolutionary features, regSNP-intron is already largely better than SPANR and CADD. This surprised me, as I believe at least SPANR uses the evolutionary features and part of the splicing features. Also, it is not likely that RF performs so much better than neural network used in SPANR on this task. 

*Response: We agree with the reviewer that the comparison of our method with SPANR may not be fully fair. This is due to the fact that SPANR aims to predict the functions of variants based on splicing outcome, while the goal of the regSNPs-intron is to evaluate the pathogenic impact of iSNVs on splicing. We discussed this difference in the original submission and we have extended it in this revised manuscript in the Discussion section (Page 17, Paragraph 2). This difference is one of the reasons why we speculate that the protein structure features is a major contributing factor in the improved performance of evaluating pathological impacts. We also agree that a better training dataset is another important factor. To avoid the potential inflation on the model performance due to the similarity of training and testing dataset, we also evaluated our model based on an independent database (ClinVar). In the revised manuscript, we extended our discussion to better address these concerns.* 

A few minor comments: 
- Fig 1: please add the number of features in each category, e.g., how many for splicing-junction score, and BRP binding score 

*Response: We have added the number of features in Figure 1.* 

- Table S1: need more description on each feature 

*Response: We have updated Table S1 and provided a more detailed description of each feature.* 

- Fig 3D & 3H: why introduce 20 bins instead use each dot for an iSNV. Here, each dot denotes a set of iSNV in a bin, but the number of iSNV in each bin can be different. One supplementary figure with the original instead of binned iSNV would help. 

*Response: We have considered reviewer’s advice and tried plotting with original iSNVs. However, there are over 600,000 iSNVs in GTEx and over 2 million iSNVs in ExAC. Plotting the original iSNVs without binning severely compromised the visual clarity. Actually, this problem was the main reason we decided to bin the iSNVs. The purpose for binning was to condense multiple iSNVs into one dot in the plot; as a result, one dot denotes the average pathogenic probability for many iSNVs. Hence this approach has reduced the dimension for data visualization and analysis.* 

- Fig 4: not sure what each dot denotes? Is it an exon for each dot? If so, why so few, as 75,119 exons are mentioned in line 11 page 12? 

*Response: Each dot denotes the average probability value of all the exons that each has a certain number of X iSNVs nearby (within ±300 bp of junction site). Values of X are shown on the x-axis. Here we have also aggregated many exons into one dot to reduce the dimension of visualization for better data presentation. In the revised manuscript, we clarified this description in the Results section (Page 13, Paragraph 2) and Figure Legends (Page 34, “Figure 4E”).* 

- In the results section for "drug-induced cytotoxicity", only the pathogenic iSNV has been reported. How about the neutral iSNVs with the similar distance to the splice junction? 

*Response: As requested by the reviewer, we have added the numbers reported for neutral iSNVs in the Results section. See Page 14, Paragraph 1.*

**Second round of review**

**Reviewer 1**

I am very disappointed that the authors spent minimal effort in the revision. Many of the issues raised previously were not addressed sufficiently.

1.     Regarding literature review of variant annotation. The authors did not cite any additional methods. This is unacceptable. I don’t understand why the authors are so resisting to acknowledging peers’ work even when given specific request.   

2.     The probability formulas and notation in pages 21 to 22 are still a complete mess. The section needs a complete overhaul with the help of a statistician. The authors need to specify what are all the parameters? What distribution does each of them follow? What are their prior distribution? What is the complete likelihood? What are the posterior distributions of the key parameters?    
•     Page 21, M=log_2 … this is not a likelihood ratio.
•     Page 22. P = … this is not a posterior probability. 

3.     The generalized linear mixed effect model shown on page 26 is still wrong. Y is a read count, integers. But the right hand side of the equation is not. How can they be equal? The liner mixed effect model should be placed on the parameters of the negative binomial distribution.

**Reviewer 2**

Thank the authors for clarification and I have a few follow-up comments.

Major 2: regarding the methods comparisons, the additional discussion helps. On the other hand, it would be informative to compare to the earlier work from the authors for other variants, e.g., regSNPs‑splicing (Zhang et al. Hum Genet, 2017), which may help evaluating the novelty of this extension. 

Minor 3: regarding the Fig 3D & 3H (current Fig 4), I agree that plotting dots may results in less clarity. However, I still think it is important to show the distribution of the minor allele frequency (MAF) of both pathogenetic and neutral variants, maybe in a supp figure. Actually, MAF is probably a big confounder on the pathogenetic prediction, namely, the iSNV with lower MAF is more likely to pathogenetic, though it could be taken as a predictive feature from a different perspective. Nevertheless, it would be interesting to explore the prediction performance after eliminating the MAF bias and see if the proposed features are still predictive.

**Authors Response**

**Point-by-point responses to the reviewers’ comments:**

Reviewer #1: I am very disappointed that the authors spent minimal effort in the revision. Many of the issues raised previously were not addressed sufficiently. 

1. Regarding literature review of variant annotation. The authors did not cite any additional methods. This is unacceptable. I don’t understand why the authors are so resisting to acknowledging peers’ work even when given specific request. 

*Response: We did not provide additional review on the non-coding variant annotation since most of the existing tools are for the variants in the enhancer/promoter regions, and do not focus on splicing analysis. We agree that the annotation and functional prioritization of these variants are important, and therefore, have provided a more comprehensive literature review of existing methods (Page 3, Paragraph 2).* 

2. The probability formulas and notation in pages 21 to 22 are still a complete mess. The section needs a complete overhaul with the help of a statistician. The authors need to specify what are all the parameters? What distribution does each of them follow? What are their prior distribution? What is the complete likelihood? What are the posterior distributions of the key parameters? 
• Page 21, M=log_2 … this is not a likelihood ratio. 
• Page 22. P = … this is not a posterior probability. 
 *Response: We apologize for confusion in the previous formulation, and consulted with a statistician to go over our terminology. Formulas and notations have been updated for clarity. Details regarding to key parameters and their distributions, as well as the process of calculation have been fully described. Meanwhile, we have also clarified the definitions and transformations of formulas. Due to the length of passage, we have moved this section to supplemental information. Please refer to the updated Supplemental Text S1 for details.* 

3. The generalized linear mixed effect model shown on page 26 is still wrong. Y is a read count, integers. But the right hand side of the equation is not. How can they be equal? The liner mixed effect model should be placed on the parameters of the negative binomial distribution. 
 *Response: By the model equation per se, the left side Y is the read count with support of the integer domain, whereas the right side is the regression which yields from negative infinity to infinity. We think the concern arises because the supports of the two sides do not match. We apologize for overlooking this error in the expression of formula. In fact, we used the expectation of Y, E(Y) from the negative binomial distribution as the outcome, with the link function of logarithm. In other words, the regression was with respect to log(E(Y)) in our actual work process. Therefore, both sides in the equation range from negative infinity to infinity so that the regression can be good. We have updated the text and equation for this part in the revised manuscript (Page 25, Paragraph 1).* 


Reviewer #2: Thank the authors for clarification and I have a few follow-up comments. 

Major 2: regarding the methods comparisons, the additional discussion helps. On the other hand, it would be informative to compare to the earlier work from the authors for other variants, e.g., regSNPs-splicing (Zhang et al. Hum Genet, 2017), which may help evaluating the novelty of this extension. 

*Response: The tool regSNPs-splicing (Zhang et al. Hum Genet, 2017) was designed to predict the pathogenicity of (synonymous) exonic single-nucleotide variants. The tool presented here, regSNPs-intron, mainly focuses on the variants in the intronic region. In addition, we present a novel experimental high-throughput reporter assay, ASSET-seq, for experimentally validated their roles in splicing regulation. This assay not only provides a validation for the computational prediction, it also offers a valuable tool for experiment-based screening on functional variants.* 

Minor 3: regarding the Fig 3D & 3H (current Fig 4), I agree that plotting dots may results in less clarity. However, I still think it is important to show the distribution of the minor allele frequency (MAF) of both pathogenetic and neutral variants, maybe in a supp figure. Actually, MAF is probably a big confounder on the pathogenetic prediction, namely, the iSNV with lower MAF is more likely to pathogenetic, though it could be taken as a predictive feature from a different perspective. Nevertheless, it would be interesting to explore the prediction performance after eliminating the MAF bias and see if the proposed features are still predictive. 

*Response: As suggested by the reviewer, we have retrieved the MAF data for all iSNVs collected from ExAC and GTEx databases. Distributions of the MAF values are shown in the new Supplementary Figure S9 (mentioned in the revised manuscript in Page 13, Paragraph 2). Pathogenic iSNVs were those with the prediction false-positive rate (FPR < 0.1, same as defined in the manuscript), and neutral iSNVs were vice versa. Since the number of neutral iSNVs (Nneutral) is greater than the number of pathogenic iSNVs (Npathog), we sampled an equal number of Npathog ones with the lowest scores of pathogenicity (i.e. probability that the iSNV causes disease) from the total Nneutral neutral iSNVs to use as the neutral class in the plot. This practice aims to avoid visual bias caused by uneven numbers of iSNVs in the respective pathogenic and neutral classes. 
Noteworthy, we did not use MAF information in the model-building, thus predictions of regSNPs-intron are not expected to be dominated/confounded by MAF. 
Key metrics for the MAF values are listed below. (A - D) are data from ExAC, and (E - H) are data from GTEx: 
(A) Alelle frequency range 0 ~ 0.25 (on-ss iSNVs): N = 87322; Pathogenic: mean = 3.57e-04, sd = 5.70e-03; Neutral: mean = 1.73e-03, sd = 1.53e-02 
(B) Alelle frequency range 0 ~ 0.25 (off-ss iSNVs): N = 244188; Pathogenic: mean = 1.05e-03, sd = 1.11e-02; Neutral: mean = 1.47e-03, sd = 1.35e-02 
(C) Alelle frequency range 0 ~ 0.5 (on-ss iSNVs): N = 87387; Pathogenic: mean = 6.28e-04, sd = 1.16e-02; Neutral: mean = 4.79e-03, sd = 3.72e-02 
(D) Alelle frequency range 0 ~ 0.5 (off-ss iSNVs): N = 245101; Pathogenic mean = 2.42e-03, sd = 2.55e-02; Neutral: mean = 3.94e-03, sd = 3.36e-02 
(E) Alelle frequency range 0 ~ 0.25 (on-ss iSNVs): N = 1417; Pathogenic: mean = 1.59e-02, sd = 3.42e-02; Neutral: mean = 4.18e-02, sd = 6.28e-02 
(F) Alelle frequency range 0 ~ 0.25 (off-ss iSNVs): N = 76609; Pathogenic: mean = 2.98e-02, sd = 5.22e-02; Neutral: mean = 3.32e-02, sd = 5.52e-02 
(G) Alelle frequency range 0 ~ 0.5 (on-ss iSNVs): N = 1457; Pathogenic: mean = 2.59e-02, sd = 6.95e-02; Neutral: mean = 9.09e-02, sd = 1.34e-01 
(H) Alelle frequency range 0 ~ 0.5 (off-ss iSNVs): N = 85017; Pathogenic: mean = 6.37e-02, sd = 1.16e-01; Neutral: mean = 7.43e-02, sd = 1.25e-01 

Overall, same as the first round review, we found the second round review is very informative, and after addressing the concerns, the manuscript is significantly improved. We thank again for the time spent by the reviewers, and look forward to the editorial decision. 
--------------------*

**Third round of review**

**Reviewer 1**

I still have follow up questions. I trust the editor will decide whether further revision is necessary. 
1. I disagree with moving the details of the model to supplementary material. They should be included in the main text with clear definition and explanation. No one will read the supplementary material and this part is important.

2. The formula on VP is incorrect. Please double check. At least a parenthesis should be placed inside the double summation  to contain the subtraction.

3. Based on the description, I think Omega(S) should be Phi(S,B)/Phi(S,NB). Why the denominator is (1-Phi(S,NB))?.

**Reviewer 2**

Both of my concerns have been addressed. Though the authors didn't provide comparison with their earlier work (with partially overlapped feature set), I agree that the novelty of this work is marginally enough for Genome Biol.

**Authors Response**

**Point-by-point responses to the reviewers’ comments:**

Reviewer #1: I still have follow up questions. I trust the editor will decide whether further revision is necessary.

1. I disagree with moving the details of the model to supplementary material. They should be included in the main text with clear definition and explanation. No one will read the supplementary material and this part is important.

*Response: According to the reviewer’s suggestion, we have moved the method descriptions in the supplementary text back into the main text. Therefore, the splicing features that constitute an important component of the algorithm, can be more clearly defined and explained. Please see in the revised manuscript (main text) from Page 21, Paragraph 2 to Page 26, Paragraph 4.*

2. The formula on VP is incorrect. Please double check. At least a parenthesis should be placed inside the double summation to contain the subtraction.

*Response: We thank the reviewer for the reminder. Now the formula has been corrected as suggested. See Page 22, Paragraph 3, the second formula, in the revised manuscript.*

3. Based on the description, I think Omega(S) should be Phi(S,B)/Phi(S,NB). Why the denominator is (1-Phi(S,NB))?

*Response: The OMEGA(S) score formula was designed in this way because we supposed that it should be more likely to observe a larger matching score to the PWM of an RBP if the specific SNV allele promotes RBP-binding; and in contrast, it should be less probable to observe a large matching score if the allele disrupts RBP-binding. In fact, this rationale had been explained in the supplemental figure and text (Supplemental Figure S10 and Text S1, last revision). On the other hand, the rationale of this formula design was developed in previous works (Zhang et al., Hum Mol Genet 2014, PMID: 24436305; Zhang et al., Hum Genet 2017, PMID: 28391525). Hence, here we have re-stated and explained the formula design more clearly in the main text with citations to supporting references. Please see in the revised manuscript (main text) at Page 24, Paragraph 2.*

Reviewer #2: Both of my concerns have been addressed. Though the authors didn't provide comparison with their earlier work (with partially overlapped feature set), I agree that the novelty of this work is marginally enough for Genome Biol.
